# An Insurance Value Modeling Approach That Captures the Wider Value of a Novel Antimicrobial to Health Systems, Patients, and the Population

**DOI:** 10.36469/001c.75206

**Published:** 2023-07-18

**Authors:** Mei S. Chan, Richard Holloway, Robert King, Rosie Polya, Rebecca Sloan, Jack C. Kowalik, Tom Ashfield, Luke S.P. Moore, Thomas Porter, Jonathan Pearson-Stuttard

**Affiliations:** 1 Health Analytics Lane Clark & Peacock LLP, London, UK; 2 Pfizer Ltd., Tadworth, UK; 3 Chelsea and Westminster Hospital NHS Foundation Trust, London, UK; 4 NIHR Health Protection Research Unit in Healthcare Associated Infections and Antimicrobial Resistance Imperial College London, London, UK; 5 North West London Pathology Imperial College Healthcare NHS Trust, London, UK; 6 Department of Epidemiology and Biostatistics Imperial College London, London, UK

**Keywords:** insurance value, wider elements of value, antimicrobials, simulation modeling, pharmacoeconomics

## Abstract

**Background:** Traditional health economic evaluations of antimicrobials currently underestimate their value to wider society. They can be supplemented by additional value elements including insurance value, which captures the value of an antimicrobial in preventing or mitigating impacts of adverse risk events. Despite being commonplace in other sectors, constituents of the impacts and approaches for estimating insurance value have not been investigated.

**Objectives:** This study assessed the insurance value of a novel gram-negative antimicrobial from operational healthcare, wider population health, productivity, and informal care perspectives.

**Methods:** A novel mixed-methods approach was used to model insurance value in the United Kingdom: (1) literature review and multidisciplinary expert workshops to identify risk events for 4 relevant scenarios: ward closures, unavoidable shortage of conventional antimicrobials, viral respiratory pandemics, and catastrophic antimicrobial resistance (AMR); (2) parameterizing mitigable costs and frequencies of risk events across perspectives and scenarios; (3) estimating insurance value through a Monte Carlo simulation model for extreme events and a dynamic disease transmission model.

**Results:** The mean insurance value across all scenarios and perspectives over 10 years in the UK was £718 million, should AMR remain unchanged, where only £134 million related to operational healthcare costs. It would be 50%-70% higher if AMR steadily increased or if a more risk-averse view (1-in-10 year downside) of future events is taken.

**Discussion:** The overall insurance value if AMR remains at current levels (a conservative projection), is over 5 times greater than insurance value from just the operational healthcare costs perspective, traditionally the sole perspective used in health budgeting. Insurance value was generally larger for nationwide or universal (catastrophic AMR, pandemic, and conventional antimicrobial shortages) rather than localized (ward closure) scenarios, across perspectives. Components of this insurance value match previously published estimates of operational costs and mortality impacts.

**Conclusions:** Insurance value of novel antimicrobials can be systematically modeled and substantially augments their traditional health economic value in normal circumstances. These approaches are generalizable to similar health interventions and form a framework for health systems and governments to capture broader value in health technology assessments, improve healthcare access, and increase resilience by planning for adverse scenarios.

## BACKGROUND

Antimicrobials have supported medical advances and saved many lives, but antimicrobial resistance (AMR) is an increasing concern at a time where novel antimicrobial discovery has slowed.^1^ Global bacterial AMR-associated deaths were estimated to be 4.95 million in 2019,^2^ while O’Neill’s AMR review projected that AMR-related deaths could rise to 10 million per year by 2050.^1^ The economic impact of AMR could also reach $100 trillion^3^ or reduce the gross domestic product (GDP) by 3.8% by 2050.^4^ Recently, COVID-19 triggered a wave of secondary bacterial infections^5^ and adversely affected health systems and wider society, with a 9.7% reduction of GDP in the United Kingdom (UK) in 2020.^6^

The UK National Institute for Health and Care Excellence (NICE) recently published guidance on evaluating the wider benefits of using novel gram-negative antimicrobials to tackle the growing threat of AMR.^7,8^ These will be made available through a subscription-style reimbursement model to reincentivize innovation and investment in antimicrobials, as traditional payment mechanisms have failed to incentivize investment.^3^

A key strategy for quantifying the value added to the health system by a health innovation and the value added by research and development of novel therapeutics is the estimation of insurance value. Insurance value is defined by the UK Office for Health Economics (OHE) as “the value of having a treatment available in case of a future major or rapidly escalating health problem.”^9^ It is a key element of value identified by the NICE AMR guidance,^7,8^ 1 of 10 elements in the OHE framework relevant to antimicrobials,^9–11^ and 1 of the 5 STEDI elements (*S*pectrum, *T*ransmission, *E*nablement, *D*iversity, and *I*nsurance^7^), where these 5 elements form the most relevant subset of the OHE framework, and is not typically included in traditional health technology assessments (HTA).^9–11^ In the AMR context, it is the value associated with avoiding potential costs to health systems and society through making a treatment such as a novel antimicrobial available, or keeping it in reserve, for a range of adverse scenarios where AMR could become substantially worse. Despite this, there is no clear methodology for estimating the insurance value of an intervention.

Definitions of insurance value focus on operational healthcare costs^9,10^ but could extend to include wider perspectives: population health impacts on patients and societal impacts. Values from these perspectives are treated as mutually exclusive and can be aggregated to capture value more holistically. A prior study has outlined a framework for how the AMR-related traditional economic value of vaccines can be quantified from both population health and societal perspectives.^12^

To estimate insurance value from projected impacts of extreme events, extreme value theory has been used in other contexts. In the non-life-insurance sector, where extreme value theory is commonly applied, separate models for (1) frequency of the event insured and (2) its severity (losses covered by insurance) are parameterized. Frequency and loss data are fitted to statistical distributions,^13^ then a Monte Carlo simulation is conducted to obtain the expected value of losses.^14^

Adding insurance value to the conventional health economic value of a hypothetical medical technology was estimated to increase its value by 38% to 62%.^15^ However, insurance value has not yet been assessed in analyses of the broader value of antimicrobials.^11^

This study aimed to assess the benefits that a novel gram-negative, broad-spectrum antimicrobial provides to health systems and society from an insurance value perspective, using a novel evidence synthesis and modeling approach.

## METHODS

A comprehensive risk assessment was conducted, which integrated evidence from a scoping review of published literature (**Online Supplementary Material, Appendix 1**) and advice from key opinion leaders (KOLs) at multidisciplinary risk workshops (**Appendix 2**). This assessment provided model parameters for frequencies and severities (costs that could be mitigated) of risk events pertaining to 4 relevant scenarios. These were used to estimate insurance value via a Monte Carlo simulation and dynamic transmission modeling.

### Scenarios and Risk Events in Scope

Four relevant scenarios and risk events (events that negatively impact health systems or wider society) pertaining to these scenarios were identified, prioritized, and characterized at the risk workshops. Risk events in scope met these criteria: (1) rare events that are beyond the scope of standard operational planning, (2) severe impact on the wider health ecosystem, (3) quantifiable impact, and (4) events that could be prevented or mitigated by the novel antimicrobial.

Insurance value was assessed over a 10-year horizon in the UK, based on characterizations of moderate and severe risk events for each scenario used to parameterize the model:

- A number of hospital wards closing down due to an outbreak of infections
  - **Moderate case**: a major ward of a regional hospital is closed to new admissions for around 6 months.
  - **Severe case:** as above, but closed for around 18 months, with secondary transmissions to other wards.
- Unavoidable shortage of conventional antimicrobials, mandating urgent use of the novel antimicrobial as a replacement antimicrobial
  - **Moderate case**: a shortage of an antimicrobial occurs, lasting for 3 months. Patients are treated with relevant, more expensive substitutes during disruption.
  - **Severe case:** as above, lasting for 6 months.
- A viral respiratory pandemic resulting in affected patients experiencing relevant secondary bacterial infections
  - **Moderate case**: seasonal respiratory viruses resulting in an increased number of hospitalizations with relevant infections.
  - **Severe case:** a severe pandemic resulting in an increased number of hospitalizations with relevant infections.
- A catastrophic AMR scenario, characterized by a large increase in AMR levels and microbial infection rates

This 10-year time horizon was used as it matches with health systems’ planning horizons (eg, the UK National Health Service Long Term Plan) and has also been used in cost-effectiveness modeling for AMR.^16^ The NICE assessment of wider benefits of using novel gram-negative antimicrobials was also conducted for a 10-year contract period, although in underlying analyses, wider value was modeled over a longer 20-year horizon.^7,8^

### Risk Assessment Based on Scoping Review and Risk Workshops

The scoping review consolidated characteristics and evidence relating to operational healthcare and wider perspectives for candidate scenarios (**Appendix 1**). Model parameters relating to these impacts (costs and frequencies) were selected in the scoping review and refined with KOL advice at the workshops, where up to 9 KOLs with expertise in healthcare management, health economics, clinical care, and microbiology discussed the characteristics and relevance of candidate scenarios and their moderate and severe case parameters in a semistructured format (**Appendices 1 and 2**). The risk assessment resulted in the detailed list of parameters and their rationale (**Appendix 3**).

Health impacts were expressed in monetary terms by multiplying quality-adjusted life-years (QALYs) lost by a value of life corresponding to the NICE willingness-to-pay threshold of £30 000 (**Appendix 1**). For the UK context, KOLs recommended using the method for estimating wider societal costs published by NICE in 2013,^17^ where relevant components of wider societal costs were paid productivity, unpaid productivity, and informal care costs. These components were also identified in the scoping review and discussed in the health economic workshop (**Appendices 1 and 2**). Modeling approaches for these components are summarized in **Appendix 1**.

In the absence of alternative characterizations of catastrophic AMR, the oft-cited O’Neill Review scenario (an immediate increase in AMR levels by 40%, remaining constant to 2050, and a corresponding instant doubling of infection rates)^1^ was recommended for analysis (**Appendices 1 and 2**). KOLs also advised using COVID-19 evidence to characterize a severe pandemic and to anticipate health systems’ responses to catastrophic AMR (**Appendix 2**).

### Monte Carlo Simulation Model

A Monte Carlo simulation model was used to model insurance value stochastically for each of the first 3 scenarios. The use of a stochastic rather than deterministic model captured uncertainty inherent in the estimates of relevant financial impacts and frequency of occurrence of risk events, while the Monte Carlo simulation approach is commonly used in insurance and financial modeling and to model expected value of losses for pandemic risk in environmental economics and global health, notably by The Lancet Commission on Investing in Health.^14^

Using the extreme value theory framework, the Poisson distribution, commonly used to describe rare event occurrence,^13^ was used to model the frequency of risk events (**Appendix 3**). The generalized Pareto distribution, commonly used to model rare risks with severe impacts,^13^ was then used to model the severity of the impacts (relevant costs per event; **Appendix 3**). Severity distributions were first used to model operational healthcare costs, then overall impacts consisting of operational and wider health impacts and the productivity and informal care aspects of societal impacts.

**Figure 1.**
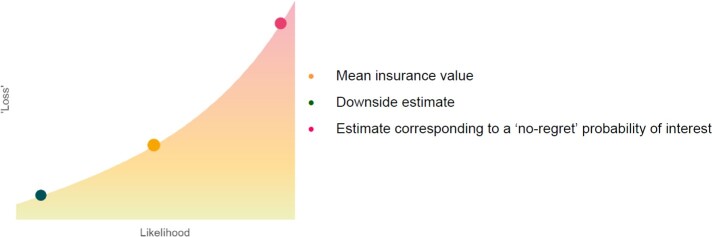
Insurance Value Estimates Output by the Monte Carlo Simulation Model, Ranked by Magnitude of Loss and Likelihood

For each scenario, to model parameter uncertainty, 2 million insurance values were simulated from the joint frequency and severity distribution over a 1-year time horizon. The probability-weighted mean insurance value (across simulations) was obtained. A 1-in-10 year downside estimate that represented the situation where the estimate was higher than 90% of the simulated values was also reported. Insurance value could also be assessed at any specified probability according to risk appetites (“no-regret” probability; **Figure 1**). This model was developed in Microsoft Excel (parameterization and plotting) and R (simulation modeling). The simulation modeling code implements a standard Monte Carlo simulation model (widely used and validated in insurance and financial modeling) and is shown in **Appendix 5**.

A 10-year time horizon was used, and 2 subscenarios were investigated: (1) main analysis, where AMR levels remained unchanged, and (2) linearly increasing AMR levels year-on-year. Present values across 10 years were obtained by projecting the simulated values for each 1-year horizon using a 2% inflation rate^18^ then discounting by the UK Treasury–recommended rate of 3.5%.^19^

### Dynamic Transmission Model for Catastrophic AMR Scenario

For the catastrophic AMR scenario, instead of the Monte Carlo model, a previously published dynamic transmission model for a novel antimicrobial was used.^16^ This built on existing methods for estimating operational costs and health impacts due to changes in AMR levels and bacterial infection rates^16^ and circumvented the need to fit a frequency distribution, as this scenario is unlikely to manifest as multiple discrete events. Furthermore, details on the validation of this model have been published in the same study.^16^

This model incorporated health-related and healthcare use parameters specific to pathogen type, indication, and treatment pathway (where the novel antimicrobial was the third-line treatment), from published literature.^16^ Infection rates and AMR levels were dynamically modeled over a 10-year horizon.^16^

AMR and bacterial infection levels were modified to those for the catastrophic AMR scenario (**Appendix 3**), while other parameters remained unmodified.^16^ The impacts were estimated by calculating differences between the model outputs (discounted healthcare costs, years of life lost, and QALYs lost) for this scenario and those for the base case.

Societal impacts for catastrophic AMR were estimated by applying NICE methods for productivity and informal care impacts.^17^ The expected insurance value was estimated by multiplying the aggregated value by the probability of catastrophic AMR occurrence within 10 years. The present value was obtained by discounting the expected value by 5 years (assuming it occurred midway on average).

### Aggregation of Risks of the Scenarios

Insurance values were summed to obtain a combined insurance value distribution across the scenarios. This assumed that the scenarios occurred independently, as each scenario is rare, and reflected that the probability-weighted occurrence and impact of one of the in-scope scenarios would not substantially reduce that of another in-scope scenario. Sensitivity analyses were conducted across a range of key severity and frequency parameters (**Appendix 4**), to assess variability in modeled results due to lack of robust evidence.

## RESULTS

### Aggregate Insurance Value of AMR

**Main analysis:** In the main analysis, the overall mean insurance value of a novel antimicrobial in the UK across all perspectives and scenarios was £718 million (**Table 1**). This assumed that AMR levels remain at current levels over the 10-year period. The wider impact on health and aspects of societal costs of £584 million contributed more to the total value (81%) than the operational healthcare cost of £134 million. Across the first 3 scenarios, the insurance value was £421 million combined while for the fourth, catastrophic AMR, the insurance value was £297 million, comprising an operational cost of £28 million and a wider impact of £269 million (**Table 1**). The 1-in-10 year downside insurance values were 54% higher than the mean value across the 3 scenarios and were highest for pandemics (70% higher at £493 million; **Table 1**). One-in-10 year downside estimates were not available for catastrophic AMR due to the different deterministic approach used.

**Table 1.** Mean (**A**) and 1-in-10 Downside (90th Percentile) (**B**) Insurance Values for the Main Analysis (Million £ Over 10 Years)^a^

**(A) Mean Insurance Values**			
	**Perspective (Million £ Over 10 Years)**		
**Scenario**	**Operational Healthcare Impact**	**Wider Impact on Health, Productivity, and Informal Care**	**Total**
Scenario 1: Ward closure	5.9	20.9	26.8
Scenario 2: Unavoidable shortage of conventional antimicrobials	96.7	7.3	104.0
Scenario 3: Viral respiratory pandemic	3.3	287.0	290.3
Scenario 4: Catastrophic AMR^b^	28.1	268.8	296.9
**Combined across scenarios**	**134.0**	**583.9**	**717.9**
**First 3 scenarios (1 to 3)**	**105.9**	**315.2**	**421.1**
			
**(B) 1-in-10 Downside (90th Percentile) Insurance Values**			
	**Perspective (Million £ Over 10 Years)**		**1-⁠in-⁠10 vs Mean (% Increase)**
**Scenario**	**Operational Healthcare Impact**	**Total (Operational Healthcare + Wider Impact on Health, Productivity, and Informal Care)**	
Scenario 1: Ward closure	11.0	45.1	69
Scenario 2: Unavoidable shortage of conventional antimicrobials	161.9	181.9	75
Scenario 3: Viral respiratory pandemic	10.7	492.9	70
**First 3 scenarios (1 to 3)**	**173.0**	**648.9**	**54**

Abbreviation: AMR, antimicrobial resistance.

Note: The 1-in-10 downside insurance values for the 3 scenarios are not additive, as the 90th percentile for the combined insurance value across scenarios does not simply correspond to the 90th percentiles of insurance values for each scenario.

^a^These data assume that AMR levels remaining at current levels. Impact is expressed as millions of pounds over 10 years.

^b^These results are reported as main analysis results even though AMR levels varied dynamically (but not catastrophically) over the 10 years.

Increasing AMR levels over the 10-year period: Insurance value was sensitive to the projection of increasing AMR levels, with the mean value across the first 3 scenarios increasing by 56% to £657 million from the value of £421 million in the main analysis (**Table 2** vs **Table 1**). The 1-in-10 year downside values displayed similar trends across these scenarios, increasing by 44% to £937 million from £649 million (**Table 2** vs **Table 1**). The largest increases were in the operational healthcare impact of conventional antimicrobial shortages and across all perspectives for pandemics, due to a 4-fold increase in patients whose treatment is disrupted and a 50% increase in patients where the novel antimicrobial is relevant, respectively (**Appendix 3**).

**Table 2.** Mean (**A**) and 1-in-10 Downside (90th Percentile) (**B**) Insurance Values for Increasing AMR Levels Case (Million £ Over 10 Years)^a^

**(A) Mean Insurance Values**			
	**Perspective (Million £ Over 10 Years)**		
**Scenario**	**Operational Healthcare Impact**	**Wider Impact on Health, Productivity, and Informal Care**	**Total**
Scenario 1: Ward closure	11.4	20.2	31.6
Scenario 2: Unavoidable shortage of conventional antimicrobials	279.0	8.5	287.5
Scenario 3: Viral respiratory pandemic	6.0	331.4	337.4
Scenario 4: Catastrophic AMR^a^	28.1	268.8	296.9
Combined across scenarios	324.5	628.9	953.4
**First 3 scenarios (1 to 3)**	**296.4**	**360.1**	**656.5**
			
**(B) 1-in-10 Downside (90th Percentile) Insurance Values**			
	**Perspective (Million £ Over 10 Years)**		**1-⁠in-⁠10 vs Mean (% Increase)**
**Scenario**	**Operational Healthcare Impact**	**Total (Operational Healthcare + Wider Impact on Health, Productivity, and Informal Care)**	
Scenario 1: Ward closure	18.1	52.5	66
Scenario 2: Unavoidable shortage of conventional antimicrobials	478.9	491.2	71
Scenario 3: Viral respiratory pandemic	13.8	552.7	64
**First 3 scenarios (1 to 3)**	496.6	936.6	43

Abbreviation: AMR, antimicrobial resistance.

Note: The 1-in-10 downside insurance values for the 3 scenarios are not additive, as the 90th percentile for the combined insurance value across scenarios does not simply correspond to the 90th percentiles of insurance values for each scenario.

^a^AMR levels varied dynamically over the 10 years rather than increasing linearly. Therefore, the same as the main analysis. results are reported in this table.

### Operational Healthcare Costs

In the main analysis, the key contributing scenario to the total operational costs of £134 million was the unavoidable shortage of conventional antimicrobials, at £97 million. Catastrophic AMR also contributed a substantial £28 million to the total cost (**Table 1**).

Conventional antimicrobial shortages were projected to affect the most patients (3000 patients per day for 90/180 days in a moderate/severe case, where 25% were relevant to the novel antimicrobial), compared with ward closures (3000/10 000 bed days lost, 20% were relevant) and pandemics (50 000/400 000 patients, where 0.8% of patients would have a relevant infection; **Appendix 3**). Catastrophic AMR had a lower likelihood of occurrence and would affect approximately 100 000 patients, where the novel antimicrobial would be relevant 80% of the time (**Appendix 3**).

**Figure 2.**
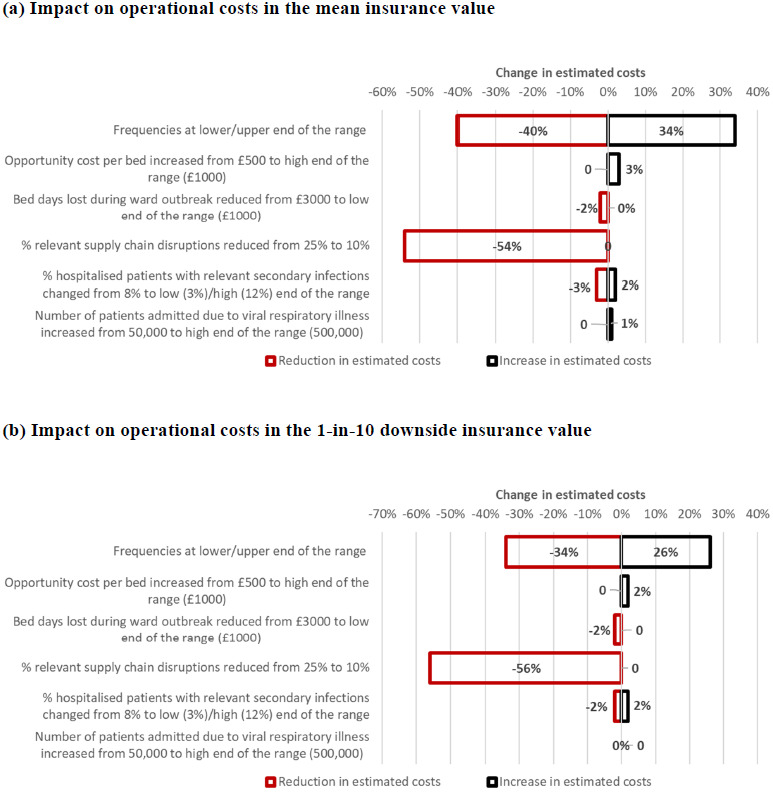
Results of Sensitivity Analyses on Operational Healthcare Costs^a^ Note: The largest changes to the estimated mean insurance value of £105.9m across these 3 scenarios were -£42.4 million/+£36.0 million for frequencies at lower/upper end of the range and -£57.2 million for relevant supply chain disruptions reduced from 25% to 10%. The largest changes to the estimated 1-in-10 downside insurance value of £173.0 million across these 3 scenarios were -£58.8 million/+£45.0 million for frequencies at lower/upper end of the range and -£96.9 million for relevant supply chain disruptions reduced from 25% to 10%. ^a^Results show the combined effect of ward closures, unavoidable shortage of conventional antimicrobials, and viral respiratory pandemics (percentage change from main analysis results).

Sensitivity analysis results for operational costs using different parameters were compared with those for the main analysis (**Figure 2**), except for catastrophic AMR, as sensitivity analyses of the underlying model have been published.^18^ Using upper and lower ends of the projected frequencies had a cumulative effect of increasing the mean operational costs by 34% and reducing them by 40%, respectively, while the effects on the 1-in-10 downside values were slightly dampened (**Figure 2; Table 3**). Reducing the proportion of relevant conventional antimicrobial shortages reduced the mean value by 54% and the 1-in-10 downside value by 56% (**Figure 2**). Other variations of parameters did not substantially change the values (changes of -3% to 3%; **Figure 2**).

**Table 3.** Frequencies at the Lower/Upper End of the Range for the 3 Scenarios

**Scenario**	**Frequency Parameter Type**	**Base Case**	**Lower End of Range**	**Upper End of Range**
Scenario l: Ward closure	Moderate cases	l event/3 y	l event/4 y	l event/2 y
	Severe cases	l event/10 y	l event/25 y	l event/5 y
Scenario 2: Unavoidable shortage of conventional antimicrobials	Moderate cases	3 events/10 y	2 events/10 y	4 events/10 y
	Severe cases	l event/10 y	l event/20 y	l event/8 y
Scenario 3: Viral respiratory pandemic	Moderate cases	l event/5 y	l event/10 y	l event/2 y
	Severe cases	l event/20 y	l event/35 y	l event/10 y

Additionally, changing the discount rate from 3.5% to 1.5% increased insurance value (both the overall value and each component) by 11.2%. Changing the inflation rate from 2% to 1% reduced insurance value (both the overall value and each component) by 4%, while changing it from 2% to 5% increased insurance value (both the overall value and each component) by 14%.

### Wider Impacts on Health, Productivity, and Informal Care

In the main analysis, the key contributing scenarios to the total health impact of £584 million were catastrophic AMR and viral respiratory pandemics, which contributed £269 million and £287 million, respectively (**Table 1**).

The majority of these impacts related to health impacts (£543 million of wider impacts of £584 million; **Table 1**). Underlying these health impacts were the QALYs lost and years of life lost (YLL). The estimated QALYs lost for pandemics were highest (3563/72 430 years for each moderate/severe risk event), followed by ward closures (34/328 years), then conventional antimicrobial shortages (0/2 years, as more expensive substitutes were assumed to be typically available; **Appendix 3**). Estimated YLLs followed a similar pattern to and were much lower than QALYs lost, except for ward closures. YLLs estimated for a single severe pandemic event was 19 200 years. To arrive at estimates of YLL and QALYs lost for catastrophic AMR (discounted values of 94 967 and 75 294 years, respectively), the dynamic transmission model projected 8500 deaths in the UK, or 91 deaths per 1000.

In the main analysis, the viral respiratory pandemic scenario had the largest impact on societal costs arising from changes in productivity and informal care, contributing £25 million to the overall impact of £42 million, based on estimated severities of £286 618/£4 298 780 for moderate/severe risk events. By contrast, for conventional antimicrobial shortages, they were £0/£2 795 151 and for ward closures £74 473/£300 866, respectively (**Appendix 4**). For catastrophic AMR, the best estimate productivity and informal care impact was £54 million; however, as its probability of occurrence was low, at 25% in 20 years (**Appendix 3**), the mean productivity and informal care impact was consequently lower at £6 million. Paid and unpaid productivity contributed approximately 92% to 98% to these aspects of societal costs, while informal care contributed the remainder.

Sensitivity analysis results for parameters relating to the health impacts were compared with results for the main analysis (**Table 4** vs **Table 1**). Assuming a worse infection profile increased mean health impacts by 0% to 223% (**Table 4**). Increasing each of infection duration and age of infection had a minimal impact on QALYs for ward closures (close to 0% increase) and conventional antimicrobial shortages (80% increase, as QALYs lost in the main analysis were also low), whereas for pandemics, they increased substantially by approximately 10 000/115 000 years for moderate/severe cases; hence, health impacts increased more substantially, by 223% (**Table 4**).

**Table 4.** Sensitivity Analysis of Mean Health Impacts (Percentage Increase from Main Analysis Results)

**Scenario**	**Sensitivity Analysis of Worse Infection Profile**	**Sensitivity Analysis of £60 000 Instead of £30 000 Value of Life**
Scenario 1: Ward closure	0%	149%
Scenario 2: Unavoidable shortage of conventional antimicrobials	80%	100%
Scenario 3: Viral respiratory pandemic	223%	100%
Scenario 4: Catastrophic AMR	88%	152%
**Combined across scenarios**	**150% (to £1356.6 million)**	127% (to £1231.0 million)

Note: The worse infection profiles were based on estimates informed by discussions with KOLs. For scenarios 1-3 they represented an increased infection duration to 42 days (upper bound vs best estimate of 14 days) and a younger age at infection of 50 years (lower bound, vs best estimate of 65 years), in normal circumstances. For the catastrophic AMR scenario, it represented AMR levels higher than current levels by 30% to 50% and 3 times the current infection rates (**Appendix 3**).

Using the value of life recommended by the HM Treasury in 2022 (£60 000 per life-year or QALY lost) instead of NICE (£30 000/QALY lost), the mean health impacts were projected to increase by between 100% to 152%, more than doubling the overall health impact in monetary terms from £584 million to £1231 million (**Table 4**). This change is broadly commensurate with the relative magnitudes of the two values of life used in the comparison.

For productivity and informal care impact parameters, few alternative estimates or narrow ranges of estimates were elicited (**Appendix 3**). Hence, only sensitivity analyses of universal parameters were conducted (**Figure 2**).

## DISCUSSION

### Key Findings

To our knowledge, this study is one of the first to develop an approach for estimating insurance value for a novel antimicrobial and to conduct this estimation in the UK context. We investigated 4 scenarios relevant to a novel gram-negative broad-spectrum antimicrobial: ward closures, unavoidable shortage of conventional antimicrobials, viral respiratory pandemics, and catastrophic AMR.

The overall insurance value of a novel antimicrobial if AMR remains at current levels (a conservative projection) is substantial, at £718 million over a 10-year period. This is over 5 times greater than insurance value just from the operational healthcare costs perspective (£134 million), which may reflect the comparative magnitude of the population-wide impact of disease transmission, productivity loss, and informal care, as opposed to impacts related to a subgroup of people projected to receive formal health care. Insurance value was generally larger for nationwide or universal (catastrophic AMR, pandemic, and conventional antimicrobial shortages) rather than localized (ward closure) scenarios across perspectives (**Table 1**). At the integrated care systems level, this would still reflect a material value for budgeting.

The main estimates were prudent, as they could increase by approximately 60% if AMR levels steadily increase over the 10 years, or by 50% if a more risk-averse view (corresponding to a 1-in-10 downside) is taken toward risk mitigation.

The largest contributors to variability in insurance value were:

- **Frequency of events:** Conventional antimicrobial shortages would occur most frequently, while pandemics and catastrophic AMR would have the most devastating health impacts (**Appendix 3**).
- **Bed days affected**: These were largest for the pandemic and catastrophic AMR scenarios, while fewer bed days were estimated for ward closures, which had localized impacts.
- **Relevance of the novel antimicrobial:** A much higher proportion of conventional antimicrobial supply chains and bed days lost due to ward closures than viral respiratory patients would be affected, dampening the effect of the large number of viral respiratory patients (**Appendix 3**).
- **Evidence from COVID-19**: COVID-19 impacts may have implicitly influenced the worst case infection rate projections for catastrophic AMR.

### Comparison With Existing Literature

While this study is the first of its kind in estimating insurance value, our findings are validated by previously published estimates of operational costs and mortality impacts. Operational costs of ward closures over 10 years were estimated to be £6 million with severe closures occurring once in 10 years (**Appendix 3**), compared with a previous estimate of a £7.8 million annual cost for a single NHS Trust.^20^ The estimate of a £27 million operational cost for a single severe supply chain disruption was similar to the cost to NHS of over £30 million for piperacillin/tazobactam (Tazocin) in 2017.^21^

A previous simulation study of projected losses due to influenza pandemics also found that the combined impact on mortality and national income was much higher than estimated income losses alone and that most of the impact was due to potential extreme pandemic events.^14^ YLL estimated for a severe pandemic (240 000 years across hospital patients, assuming 8% of them had secondary infections; **Appendix 3**) was comparable to published national estimates for YLLs due to COVID-19 of approximately 150 000 years in a hospital setting (assuming 19.3% of all deaths due to COVID-19 were in hospitals^22^) and 800 000 years across all settings.^23^ The modeled catastrophic AMR scenario projected 91 deaths per 1000 over 10 years, which was slightly higher than the O’Neill Review estimates of roughly 50 to 60 deaths (5-6 deaths per year).^1^ These estimates are in stark contrast to the most recent global estimate of 6.4 deaths associated with AMR for 2019.^2^

### Strengths and Limitations

This study consolidated extensive evidence through a comprehensive scoping review and detailed multidisciplinary discussions with experts. This was crucial to identify plausible parameters for this unique analysis. A holistic methodology that considered wider population health perspectives and key aspects of societal cost, frequency of these risk events, parameter uncertainty, and nontraditional but substantial operational costs (**Appendix 3**) was applied.

The modeling approach was improved through Monte Carlo simulations, which incorporated stochastic uncertainty in both the frequency and severity of adverse events.^14^ The range of estimates due to uncertainty in risk assessments and model parameters were captured in simulated insurance values. While these were summarized into mean and 1-in-10 downside values, other summary values could be easily calculated to aid decision making at any risk appetite.

The aspects of societal cost analyzed in this study included the key paid and unpaid productivity and informal care impacts but did not include other impacts (eg, private paid and unpaid consumption and government consumption).^17^ The potential for double-counting the component societal and health impacts (both of which are dependent on QALYs; **Appendix 2**) was limited for 3 reasons. First, the NICE societal impact model^17^ accounts for productivity and informal care impacts from immediate changes in time spent in hospital and waiting to be admitted while ill, while these health impacts were driven by longer-term changes in health (eg, changes in mortality profiles; **Appendix 1**). Quality-of-life case mixes were proxies for productivity levels and informal care need rather than measures of health, unlike approaches where long-term mortality impact was treated as a productivity impact.^24^ Second, analysis of the QALY-productivity loss overlap (using EQ-5D) found that EQ-5D inherently accounted for negligible productivity loss.^25^ Finally, societal impacts are usually treated as additive to elements in traditional health economic analyses.^9,10^

While the detailed evidence synthesis and model parameters were highly relevant to the UK setting, this may have limited the generalizability of the study findings to HTA bodies and health systems that differ from those in the UK. On the other hand, the modeling approaches used are generalizable to any setting.

The Monte Carlo and dynamic transmission models had the following limitations:

- As with any mathematical and statistical model, the results of the Monte Carlo and dynamic transmission models depend on parameter assumptions (listed in **Appendix 3** for this study) and the choice of the populations that inform these assumptions.
- Since these scenarios are rare, there is limited evidence on the frequency and severity of losses and differential impacts between healthcare facilities. The comprehensive risk assessment informed the characterization and parameterization of relevant risk events.
- The possibility that one risk event is causally linked to another was not accounted for. The Monte Carlo model can capture correlations; however, there was insufficient evidence on these scenarios to develop this correlation structure, and allowing for interdependence would not affect mean values.
- Unlike the Monte Carlo model, the dynamic transmission model is deterministic and did not capture statistical uncertainty.^16^ Parameter variability could only be investigated via sensitivity analysis.

There is no known approach for valuing the knock-on impact of novel antimicrobial use on its longer-term resistance or effectiveness levels; hence, this is not accounted for in insurance value estimates. However, the impact on insurance value due to projected increases in resistance levels for the existing antimicrobials has been assessed. Further research on estimating potential adjustments to the insurance value to allow for the long-term impacts of utilizing novel antimicrobials on their own and other antimicrobials’ resistance levels (where the increases in resistance levels for existing antimicrobials is not treated as a benefit) is recommended.

Addressing these limitations is likely on balance to result in increased rather than reduced overall insurance value estimates. Since many parameters were required, a range of sensitivity analyses were conducted to mitigate parameter uncertainty.

### Additional Sources of Value

The sources of value for a novel antimicrobial in this study spanned a wider range of perspectives and its potential to mitigate multiple adverse scenarios, while only operational healthcare costs and health impacts under normal circumstances are considered in traditional HTAs.

Nevertheless, the estimated insurance value is likely to be substantially lower than the actual value (and thus prudent) for 4 reasons:

- The insurance value is only one of the 10 elements of value of novel antimicrobials identified by OHE, and other elements add substantially to financial, societal, and health benefits of novel antimicrobials. Previous literature has suggested elements are additive, and therefore the insurance value would supplement a traditional health economic evaluation of its value to the healthcare system. The potential for overlaps between elements (and whether in-scope situations constitute adverse scenarios), such as increased spreading of infection contributing to both insurance value and transmission value, or insurance vs enablement value of ward closures, has not been discussed.
- For the first 3 scenarios, the main estimates assumed that AMR remains at current levels over 10 years. If AMR levels increased gradually (despite increasing AMR stewardship^26,27^), insurance value would be 70% higher (**Table 2** vs **Table 1**).
- Four adverse scenarios were analyzed, but additional scenarios may further characterize broader risk mitigation provided by novel antimicrobials.
- For the health impacts, monetary value was obtained using the NICE threshold of £30 000 per QALY. This threshold varies between countries according to prevailing risk appetites, while values attributed to health across UK government departments are inconsistent.^28^ Using the value of life recommended by the HM Treasury in 2022 (£60 000 per life-year or QALY lost) instead of NICE (£30 000 per QALY lost) was estimated to slightly more than double the mean health impacts (**Table 4**). For major catastrophes, higher values were suggested for health impacts.^10,11^ COVID-19 may have increased awareness of risk-averse scenarios (**Appendix 2**) and willingness to invest in mitigating other public health crises.
- This insurance value was projected for a 10-year horizon, and would double if a 20-year horizon was used for planning or resource allocation, or increase to 2.5 times for a 30-year horizon. In fact, NICE proposed in its assessment of wider benefits of using novel gram-negative antimicrobials that 60% to 100% of the benefits estimated over a 20-year horizon should be assigned to a 10-year contract period.^7,8^

### Implications

This study assessed the insurance value of a novel antimicrobial, but these approaches are generalizable to similar health interventions, such as holding medicines and vaccines in reserve for pandemic preparedness. Adoption of the insurance value framework by both HTA agencies and industry would more comprehensively attribute value added to the health system and encourage research and development of novel therapeutics. For communicable diseases in particular, antimicrobials are a common good, and therefore insurance valuations capturing wider population health and societal perspectives are especially relevant.

Since insurance value can be estimated at different risk levels and for different time horizons, these findings can help health systems, governments, and businesses think critically about their risk appetites and plan for adverse events, with health becoming a cornerstone of risk mitigation plans. If time horizons beyond 10 years for these plans were more appropriate (eg, due to long-lasting impacts of catastrophic AMR and pandemics), insurance values over these longer time horizons are substantially higher than the 10-year insurance values reported in this study.

This analysis advances the methodology for assessing the broader value of health interventions, through adapting and supplementing models for risk management of extreme events in insurance and other sectors. The resulting estimates account for the likelihood of relevant risk events and parameter uncertainty. This study also supplements the limited evidence base on broader value by characterizing scenarios, risk events, and perspectives relevant to a novel antimicrobial. It enhances recent recommendations to conduct and embed broader value assessments in health economic evaluations.^9–11,15,16,29,30^

## CONCLUSION

Our analysis demonstrates that the substantial and comprehensive insurance value of a novel antimicrobial can be systematically modeled and incorporated into a HTA or evaluation framework. The economic, health, and societal value of making a novel antimicrobial available, or holding it in reserve, should be reflected by augmenting its traditional health economic value with its insurance value and exploring additional elements of value. This ties in with increasing attention given to preventing future health threats^31^ and recognizing the wider value of therapeutics,^9–11,16,29,30^ where health is valued as an asset rather than a burden.^32^ Increased awareness and data collection would ameliorate limited evidence on the frequency and severity of these future impacts (due to the rarity of relevant scenarios) and variation among health systems and healthcare facilities. Incorporating more holistic value frameworks would also realign incentives around patient value and population health across the healthcare ecosystem, to spur investment and innovation.

## Supplementary Material

Online Supplementary Material
