# Supplementary material for: An Insurance Value Modeling Approach That Captures the Wider Value of a Novel Antimicrobial to Health Systems, Patients, and the Population
Source: J Health Econ Outcomes Res. 2023 Jul 18;10(2):1–9. doi: 10.36469/001c.75206 (PMC10359113; doi:10.36469/001c.75206)
Supplement: Online Supplementary Material [file jheor_2023_10_2_75206_172649.pdf]

Journal of Health Economics and Outcomes Research

Online Supplementary Material

An Insurance Value Modeling Approach That Captures the Wider Value of a Novel Antimicrobial to Health Systems, Patients, and the Population. *JHEOR*. 2023;10(2)1-9. doi:10.36469/jheor.2023.75206

Appendix 1: Scoping Review 2

Appendix 2: Risk Workshops With Key Opinion Leaders 9

Appendix 3: Model Parameters Based on Findings from Scoping Review and Workshops 14

Appendix 4: Estimating the Frequency and Severity of Risk Events for Each Scenario 27

Appendix 5: R Code for Monte Carlo Simulation 31

This supplementary material has been provided by the authors to give readers additional information about their work.

This is an open-access article distributed under the terms of the Creative Commons Attribution 4.0 International License (CCBY-4.0). View this license's legal deed at http://creativecommons.org/licenses/by/4.0 and legal code at http://creativecommons.org/licenses/by/4.0/legalcode for more information.

Appendix 1 –Scoping review

Scoping review methods

The scoping review consolidated characteristics and evidence relating to impacts from the three component perspectives for in-scope scenarios. Key search terms were: “ward closure”, “supply chain disruption”, “antimicrobial shortage”, “influenza”, “viral respiratory pandemic”, “AMR”, “drug resistance” and “secondary infection”. All study types, publications from 2006 onwards and studies based in any country were eligible, but the review considered whether the evidence related to the UK context. Ranges and central estimates of parameters were recorded (Appendix 2 and 3).

Additional rapid reviews were carried out, to identify methods for modelling societal impacts, using current NICE guidance on estimating wider societal benefits as a starting point [1], and to characterise catastrophic AMR scenarios in terms of AMR levels and infection rates. These reviews provided a basis for discussing and estimating model parameters at the workshops.

Evidence for estimating operational healthcare costs

Scenario 1: Ward closures

The frequency and severity of ward closures were parameterised in terms of i) length of closure, ii) number of closures per year and iii) cost per lost bed day.

The Wong et al systematic review into ward closures noted that there was no universal definition of ward closure and that the term was often used as part of a broader bundle of infection control measures [2]. Among studies that reported a length of closure, these lengths ranged from 3 days to 1 month [2]. Hansen et al analysed closures of medical departments using the Outbreak Database, which contains data on 1561 nosocomial outbreaks over a 40-year period, and found that the median closure time was 14 days [3] (roughly the mid-point of the systematic review’s stated range [2]). The total number of closures per year for all nosocomial outbreaks was 4.85 while for K. Pneumoniae-specific outbreaks, the rate was much lower at 0.25 closures per year [3].

Other studies into the length of ward closures had substantially higher central estimates and upper bounds. Halaby et al observed an average ward closure length of 4-months and closures per year of 0-0.27, from only two ward closures [4]. Decraene et al’s investigation of K. Pneumoniae Carbapenemase (KPC)-producing E. Coli outbreaks in Manchester found that closure length ranged from 5 days to 4.8 months, with the longest closure due to a plumbing upgrade [5].

The cost of ward closures was considered on a per year basis and in terms of cost per lost bed day. Decraene et al observed the highest costs of ward closures during an infection outbreak lasting 8 months, with an equivalent annual cost per year of £7.8 million [5]. The costs recorded by Frakking et al and Otter et al were £0.86 million [6] and £1.14 million [7] per year, respectively. Frakking et al found that the largest costs arising from the vancomycin-resistant E. faecium (VRE) outbreak were associated with the laboratory (40%), followed by nursing wards (30%), cleaning (15%) and infection control (15%) [6]. In Otter et al’s study of costs associated with an outbreak of carbapenemase-producing Enterobacteriaceae (CPE), costs were split into actual expenditure and opportunity cost, with opportunity costs further divided into productivity loss for healthcare staff, missed revenue from reduced capacity to be able to perform revenue producing procedures and costs of an extended length of stay [7]. Opportunity cost accounted for around 75% of the overall cost. Otter et al also found that the cost per lost bed day was £174 [7] (using a conversion rate of €1 = £0.86).

Scenario 2: Unavoidable shortage of conventional antimicrobials

The key inputs to parameterise conventional antimicrobial shortage include: i) length of shortage, ii) cost of price increases and iii) the number of patients affected. Several shortages of commonly used antimicrobials have occurred in the last decade, including to Tazocin (generic name: piperacillin/tazobactam) and Zerbaxa (generic name: ceftolozane/tazobactam). The frequency of disruptions appeared to be increasing in Europe, despite the region’s position as the second largest manufacturer of antimicrobials [8].

For some antimicrobials, the supply chain is so fragile that it can disintegrate due to the failure or exit of just one factory or manufacturer [9]. In 2017, an explosion in a factory, the only producer of the main active ingredient of Tazocin, resulted in a shortage of approximately a year and resulted in temporary price increases that cost the NHS more than £30 million [10]. Tazocin production was also disrupted in 2014 for 3-6 months in the US, due to manufacturing problems, and an increase in C difficile infections was seen [11]. Gross reported that there was an adjusted relative risk (RR) of 1.03 in a network of 88 hospitals in the United States that experienced a Tazocin shortage, and 1.30 in the 72 hospitals that both experienced Tazocin shortage and shifted towards use of antimicrobials considered high risk for *C. difficile* infections [11].

During the same Tazocin shortage period, a quasi-experimental study was conducted into the impact of the shortage on meropenem use. It found that there was a 111% increase in meropenem prescribing but this increase was not associated with changes in mortality, length of stay or meropenem costs [12].

A Zerbaxa shortage due to unsterile vials commenced in December 2020 and supply was estimated to resume in January 2022 (a 1-year disruption) [13]. Over 3,701 patients were treated with this drug between 2009 and 2020 (11.4 years) [13]. If treatment duration is assumed to be 10 days, this level of disruption would affect around 10 patients per day.

Shortages may occur more commonly in some countries than others. Cederwall et al postulates that shortages are common in Sweden because of the small size of its market and its “ineffective purchasing system” [14], after investigating the cost a shortage of Vancomycin and Piperacillin/Tazobactam in Sweden that affected approximately 15-20 patients per day for Vancomycin and 1,000 per day for Piperacillin/Tazobactam [14]. Costs were calculated as the sum of the additional labour required to deal with shortages along with the costs of alternative medicines [14]. The fixed cost of the shortages was £136,000, while per day costs were up to £17,255 for Vancomycin and up to £78,510 for Piperacillin/Tazobactam (using a conversion rate of 1 SEK = 0.085 GBP) [14].

Other shortages that occurred in the last 10 years were for: clindamycin, co-trimoxazole, metronidazole, co-amoxiclav, erythomycin and azithromycin [9, 15]. These antimicrobials are used in a range of surgical procedures and used to treat a range of bacterial infections.

Potential future shortages have also been considered. Due to precarious supply chains, a wide range of additional types of antimicrobials are at risk of shortage. The Access to Medicines Foundation identified 8 types of antimicrobials that are at high risk of shortage: Piperacillin-tazobactam, Ampicillin-sulbactam, Meropenem, Cafotaxime, Cefepime, Benzathine penicillin G, Gentamicin and Trimethoprim/sulfamethoxazole [9].

Scenario 3: Viral respiratory pandemics

The frequency and severity of past influenza pandemics (the predominant type of viral respiratory pandemics) were well-documented and were generally parameterised in terms of annual risk, mortality, clinical attack rate, case fatality rate, secondary infections and impact on GDP and income. However, there was little evidence of their impact on healthcare costs. Health-related characteristics of these pandemics could be used to parameterise the impact on healthcare costs and are therefore summarised here, however these characteristics are also relevant to the estimation of health impacts.

Different types of influenza have caused pandemics and epidemics at varying frequencies. A study of historic pandemic and epidemic influenza found 12 pandemics caused by influenza A and fewer pandemics from influenzas B and C in the past 300 years [16]. The frequency of influenza outbreaks vary by severity as well as by type. Madhav et al estimated that the annual risk of a severe influenza pandemic is 1%, with a severe event defined as causing 6 million pneumonia and influenza deaths globally [17]. For context, the 1918 influenza pandemic caused an estimated 20-100 million deaths globally [17].

Secondary infections are common during viral respiratory pandemics, and vary by setting and illness [18]. In the community setting among those with mild illness, 0-1% had secondary infections, while 0.6-46% of those that were hospitalised with influenza had secondary bacterial pneumonia. While among patients with influenza who had respiratory failure, required ICU or who suffered fatal infections, the incidence of bacterial lung infections ranged from 28 to 55% [18]. A systematic review of bacterial co-infection rates during the 2009 H1N1 outbreak found that the rate for patients admitted to ICU with H1N1 was 1-43% (mean: 19%), and the rate for patients hospitalised but not admitted to ICU with influenza was 1.6-76% (mean: 12%) [19].

A study of hospitalised patients in Malaysia with confirmed H1N1 infections found a rate of bacterial co-infection of 28% and also noted some viral co-infection among these patients (6%) [20]. An analysis of post-mortem samples from the 1918 influenza pandemic found that 95% of them had some evidence of bacterial infection complications [21].

Patients may be over-prescribed antimicrobials during pandemics. A rapid review of bacterial and fungal co-infection found that among those with COVID-19, 8% had bacterial or fungal co-infection during hospital admission, but 72% received antibacterial therapy. Of the patients admitted without COVID-19, bacterial or fungal co-infection was also relatively low at 11% [22].

Scenario 4: Catastrophic antimicrobial resistance (AMR)

Discussions of a catastrophic AMR scenario in a large number of AMR-related papers and reports, tend to cite the AMR scenario first proposed by the O’Neill review in 2014 - deaths from drug-resistant infections are projected to rise to 10 million per year globally by 2050, totalling 400 million by 2050, if no action is taken [23]. These projections appeared to be for a specific scenario analysed by KPMG for the O’Neill report [24], where absolute AMR levels sharply increased by 40% while infection rates for 3 kinds of bacteria (3GCR E. coli and K. pneumoniae and methicillin-resistant S. aureus (MRSA)) sharply doubled, then remained

stable until 2050 [25]. This scenario was originally intended as an 'increasing AMR' rather than 'catastrophic' scenario [23], however the sharp and substantial increases in AMR levels and infection rates suggest that it describes a more extreme scenario among possible scenarios of 'increasing AMR'. The absence of empirical support for the selection of these AMR levels and infections rates, and numerous aspects of uncertainty in these estimates were highlighted by de Kraker et al [24]. de Kraker et al did not identify any other projections of AMR burden, catastrophic or otherwise.

In addition to this main scenario, the underlying analyses by both KPMG and RAND for the O'Neill review also considered more adverse scenarios where AMR levels were projected to increase to 100% initially and remain at 100%, while infection rates for these bacteria also sharply doubled [23].

The World Bank projected economic impacts of adverse AMR scenarios, however the AMR levels that corresponded to these low and high scenarios were not reported, and neither were any health impacts for the scenarios.

Another instance of a catastrophic AMR scenario was described the UK National Risk Register [26]. The latest version, published in 2020, projected that an AMR event would have between a 1 in 5-year to 1 in 100-year likelihood of occurrence [26]. No other risk register discusses AMR as a potential catastrophe, crisis, disaster or risk, except the World Economic Forum 2013 and 2018 Global Risk Reports [27]. The 2018 Global Risk Report reported that the spread of infectious diseases (primarily as a result of AMR) was perceived as slightly less likely but with a greater impact than the average across all global risks, by survey respondents [27]. No information was provided on the characteristics that would constitute a catastrophic AMR scenario [27].

Much of the literature on AMR describes the continuation of recent trends in AMR levels in the absence of novel antimicrobials as a crisis, and therefore focus on describing the relatively risk-neutral current or recent levels of AMR. The difficulty in characterising a catastrophic AMR scenario is also echoed by Viens and Littman, who described AMR as a 'slowly emerging disaster'[28]. Viens and Littman proposed that it would be difficult to pinpoint the time of initiation and the characteristics of an AMR 'disaster', due to its gradual onset, and therefore difficult to time the response [28]. They also raised the possibility that an AMR disaster may never be treated as a disaster, due to the progressive perception of gradually-increasing AMR levels as 'normal' [28].

Antimicrobial resistance (AMR) levels in normal circumstances

In comparison, estimates of worldwide AMR levels in normal circumstances estimated that AMR levels were above 50% in some cases. Alvarez-Uria et al estimated the AMR levels in 2015 to be 64.5% for 3rd-generation cephalosporin-resistant (3GCR) E. coli, 5.8% for carbapenem-resistant (CR) E. coli, 66.9% for 3GCR K. pneumoniae, and 23.4% for CR K. pneumoniae [29]. When the AMR level was projected to 2030 these rates increased for all pathogens apart from 3GCR K. pneumoniae [29]. The projected AMR levels in 2030 were 77% for 3GCR E. coli, 11.8% for CR E. coli, 58.2% for 3GCR K. pneumoniae, and 52.8% for CR K. pneumoniae [29]. Even though their analysis did not aim to characterise a catastrophic AMR event, they proposed that their risk-neutral projections for 3GCR E. coli and CR K. pneumoniae would be 'devastating for health systems' [29]. Another study projected AMR levels of 8.0% at 2030 for CR P. aeruginosa in the UK [30].

The change in AMR over time is linked to the quantities of antimicrobial used. Durham et al projected the increase in resistance for countries according to the amount of fluoroquinolone consumed. In the high consuming countries, the rate of fluoroquinolone-resistant *E. coli* resistance was projected to be 45%, whereas in the low consuming countries, the resistance level was 33% [31]. Colson et al used structured expert judgement to quantify future levels of resistance for pathogen-antimicrobial combinations in four European countries [32]. The experts concluded that resistance for five pathogen-antimicrobial pairs (E. coli and K. pneumoniae resistant to 3rd-generation cephalosporins and carbapenems, and MRSA) would remain below 50% in 2026 [32]. These judgements were based on their assumption that antimicrobial stewardship efforts would be sustained and impactful [32].

Evidence for estimating health impacts

The health impacts in scope for this project are years of life lost and QALYs lost, estimated using information on changes in mortality profiles, infection rates and quality of life over the patients' remaining lifetime, due to the occurrence of each scenario.

Health impacts were expressed in monetary terms by multiplying QALYs lost by a value of QALY, which in the UK generally relates to the NICE willingness to pay threshold of £30,000 [33, 34]. Alternative values of QALYs have also been used, such as the HM Treasury value of a statistical life of £60,000 per QALY or per life years lost [33, 35]. A recent analysis of the value of social care outcomes in England reported its key results on both the £30,000 and £60,000 per QALY bases [36].

Evidence on changes in mortality profiles and infection rates in the shorter term were found for all scenarios, while no details on changes in quality of life or longer term non-fatal health outcomes were identified in the review for any scenario. While for overall health impacts, no evidence was found for any of the scenarios. Outside of these scenarios, Bartsch et al projected that QALYs lost

due to CR Enterobacteriaceae (CRE) infection in the US was 45,261 years, based on a hypothetical CRE incidence of 15 per 100,000 hospital patients and using a Monte Carlo simulation model [37].

Scenario 1: Ward Closure

The Wong et al systematic review of ward closures in acute care settings identified 34 eligible studies that reported the proportion of patients who died during ward closure [2]. Studies that reported the proportion in a closed ward who died gave estimates of between 4% and 85%, with only study of >50 participants reporting an estimate of 4% [2]. All studies only related to the event ward closure, with no controlled comparison event, and there were no details on each study's age distribution of participants and deaths.

The same systematic review [2] identified 3 studies (Stone et al [38], Farrington et al [39] and Selkon et al [40]) that reported changes in MRSA infection rates due to ward closure. These 3 studies reported that, respectively: (1) moving from ward closure and national guidelines to hand hygiene, education/communication, restriction of antimicrobial treatment led to reduction in incidence from 3.95 to 1.94 cases per 100 admissions in 66 wards [38]; (2) moving from MRSA screening upon ICU admission, isolation, ward closure and disinfection to a relaxed closure/reopen procedure and screening criteria resulted in increased cases from 1-2 to 74 cases in 1000 beds [39]; (3) moving from ward closure and standard barrier nursing methods to limited transfer and an isolation unit with control ventilation reduced incidence rates from 6.57 to 5.08 cases per 1000 admissions in 1000 beds [40].

Scenario 2: Unavoidable shortage of conventional antimicrobials

A meta-analysis of the impact of delayed appropriate antibacterial therapy on patients with severe bacterial infections by Zasowski et al found 19 studies that provided data on mortality outcomes [41]. Mortality was significantly lower in patients receiving appropriate therapy without delay compared with those experiencing delay (odds ratio (OR) for delay vs no delay: 0.57; 95% CI, 0.45-0.72). Mortality was also lower in the no-delay group compared with the delay group in subgroups of studies reporting mortality during ICU stay, or in patients with bacteremia (OR: 0.47 [95% CI, 0.27-0.80]; and OR: 0.54 [95% CI, 0.40-0.75]) [41]. However, Zasowski et al found no difference in the duration to appropriate therapy between those who died and those who survived (p=0.09) in the 7 studies that analysed this duration, but the heterogeneity between these studies was high (I^2=89%) [41].

Estimates of changes in infection rate due to supply chain disruption during the Tazocin shortage in 2014-2016 were reported by the Gross et al study of a network of 88 hospitals in the United States [11]. All 88 hospitals experienced Tazocin shortage and 72 of them shifted toward increased use of high-risk antimicrobials [11]. The adjusted relative risk (RR) of hospital-onset C. difficile infection for all 88 hospitals was 1.03 [95% confidence interval [CI], .85–1.26; p=0.73], while the adjusted RR for the 72 hospitals that both experienced a shortage and increased their use of high-risk antimicrobials was 1.30 [95% CI: 1.03–1.64; p<0.05] [11].

Scenario 3: Viral respiratory pandemics

The frequency, clinical attack rates and secondary infection rates related to influenza pandemics were crucial for parameterising the impact of these pandemics on operational costs to the NHS through the numbers of affected patients, and therefore summarised in Appendix 3. These characteristics of influenza pandemics are also directly relevant to the estimation of their health impacts.

Additionally, mortality was a commonly-measured health indicator for influenza pandemics. Mortality varied substantially according to the strain of influenza. Yang et al found that all-cause hospital mortality among patients with H7N9 infection was 28.9% (24/83) [42]. This was much higher than that from seasonal influenza-associated pneumonia at 4.4% but lower than that due to highly pathogenic avian influenza A (H5N1) viruses at 59% [42].

The Scientific Pandemic Influenza Group identified and assessed four influenza pandemics in the UK between 1900–2017 (in 1918-19, 1957-58, 1968-70 and 2009) and found a clinical attack rate (proportion of the population that contracted influenza during the pandemic) ranging from 35% to 5%, with the highest rate from the 1968-69 pandemic and the lowest from the 2009 H1N1 pandemic [43]. The case fatality rate in the pandemics also varied substantially between pandemics, the highest case fatality rate being 2% during the 1918-19 pandemic, while the lowest being 0.01% during the 1957-58 pandemic [43].

The mortality impact of pandemics was also estimated in terms of excess mortality. Fan et al estimated excess mortality from historic influenza pandemic data and found that six pandemics in this period had rates ranging between 0.03 and 0.08%, but that the 1918 influenza pandemic had a much higher rate of 1.1% [44]. Fan et al used parameters of 0.93 standardised mortality units (deaths per 10,000 per year) to model severe pandemics and 0.05 to model moderate pandemics [44]. For the UK population of 60 million, these correspond to estimated excess deaths of around 5500 in severe pandemics and 300 in moderate pandemics.

Scenario 4: Catastrophic antimicrobial resistance (AMR)

A commonly-cited estimate from the O'Neill review is that deaths from drug-resistant infections are projected to rise to 10 million per year globally by 2050, totalling 400 million by 2050, if no action is taken on AMR [23]. In the underlying analyses by both KPMG and RAND for the O'Neill review, where the adverse scenarios of AMR levels increasing by 40% initially and remaining constant to 2050, and increasing to 100% initially and remaining at 100%, the cumulative reductions in the world population were estimated to be 350 million and 700 million respectively [23].

The O'Neill review catastrophic scenario may already have materialised in the last few years, for example in young children hospitalised for pneumonia in 2014-2017 in Bangladesh [45]. Resistance to all routinely used empiric antimicrobials was observed in 20 of the 108 children with bacteremia (18.5%), while deaths from infections occurred in 31 of these children (28.7%) [45].

The UK National Risk Register projected health impacts of 41 to 200 fatalities in the UK [26]. The Risk Register also noted that there was a 9% increase in deaths caused by drug-resistant infections between 2017 and 2018 [26].

Estimation of societal impacts

There are several methods for estimating societal impact, which differ by the components included and by methods for estimating each component [46]. Guidance attempting to address variation on measuring societal impact has been published by NICE for the net production of patients [1], which includes formal care, informal care and unpaid production, in addition to paid production.

Productivity loss

The NICE 2013 methods for calculating productivity (termed as the 'paid' and 'unpaid' production' components) is determined by a set of intermediate inputs that define a patient profile and used the human capital approach [47]. For paid production, the intermediate inputs of age and quality of life (QoL) are used to calculate a productivity rate, or the proportion of normal working time the patient can be expected to be in paid employment. Productivity rate is then applied to the time in which a person is able (or unable in case the of productivity loss) to work and multiplied by a gross average wage, then uplifted to reflect overhead expenses associated with employment [48].

The NICE 2013 methods recommends estimating unpaid productivity from three additive components: (1) general unpaid productivity, (2) unpaid sickness care production and (3) unpaid childcare production [47].

The KPMG report underlying the O'Neill review used an alternative method that captured the societal impact of increasing AMR rates through changes in productivity and the labour force [25]. Productivity was modelled by considering 5 productivity drivers across the 156 countries: macroeconomic stability, openness to catch up in technology and best practice, quality of infrastructure, human capital and the strength of public institutions [25]. Projected impact to the labour force to 2050 was modelled by projecting the working age population, then applying the mortality rates associated with each of 4 adverse AMR scenarios [25].

Informal care costs

A model to estimate informal care requirements in the UK, which used hospital patient data on days of informal care needed according to a patient profile by age, sex, QoL and health condition (by ICD chapter) [1], was utilised by the NICE 2013 methods report [47]. The days of informal care required for a patient profile were then multiplied by the hours of care needed per day and the national average net hourly wage [47]. A reduction factor for the probability that a patient requires residential care was applied for those over 75 years, as they were assumed to receive more formal than informal care [47].

A Personal Social Services Research Unit (PSSRU) study that estimated informal care costs found that for every additional £1 spent on formal care, £0.33 was saved on informal care [49].

Evidence for estimating societal impacts

Estimates of key aspects of societal impacts, productivity and informal care, for each scenario in the study presented here follow those outlined in the NICE 2013 methods [1]. Therefore patient profiles by age, sex, QoL, ICD chapter distributions, cost multipliers and incremental time were needed for each scenario. Additional evidence that was relevant to each specific scenario is also explored here.

A project on methods to estimate the NICE cost effectiveness threshold synthesised a profile of patients admitted to hospital in England per year, using estimates of age, sex and QoL scores by ICD code [50]. Age and sex distribution of patients in each ICD code were estimated using disease prevalence from a Global Burden of Disease (GBD) study and adjusted using the risk population in England [51]. Average QoL scores per ICD code were estimated using the HODaR dataset and Medical Expenditure Panel Survey (MEPS) data, which both supplied EQ-5D by ICD code [51]. Rowen et al also summarised a general inpatient population by primary ICD chapter [47]. Both these patient profiles did not consider the pattern of comorbidities of patients.

For productivity loss estimation using the NICE 2013 method [47], a gross weekly wage was required and was £576 in June 2021 in Great Britain [52]. Informal care, however, utilised net wages, where net hourly wages were £11.55 in June 2021 [52], and assumed a 40-hour work week [47].

Scenario 1: Ward Closures

The cost effectiveness of acute ward closure due to norovirus outbreak in a UK NHS setting considered bed days lost due to the ward closure and lost productivity of healthcare staff due to sickness [53]. Beds lost per day of closure to new admissions was 3.6 days. Productivity loss due to absence of staff was £159,366-£351,213, which was 28%-35% of the total cost of a ward closure. The inclusion of the staff absence component in societal costs is debated, as it could be treated as a cost to the NHS instead [54].

Scenario 2: Unavoidable shortage of conventional antimicrobials

Two studies that investigated the effect of an antimicrobial shortage on bed days lost found that length of stay decreased during the shortage [55, 56]. Barber et al compared patients receiving meropenem as an alternative to piperacillin-tazobactam, and found that length of stay was 3 days longer in the pre-shortage group [55]. Bosso et al found that this length of stay decreased by 1.7 days [56]. Neither study explored how additional length of stay impacted costs in terms of productivity or informal care.

Scenario 3: Viral respiratory pandemics

A World Bank report estimated that Gross Domestic Product (GDP) could reduce by almost 5% in the context of a severe pandemic like the 1918 influenza pandemic, while comparatively mild pandemics such as the 2009 H1N1 pandemic may have economic impacts of less than 0.5% [57]. The economic impact has also been quantified in terms of income. Fan estimated that in the case of an extremely severe pandemic, gross national income could reduce by 12% worldwide [44]. In the same study, the mean income loss per year across all severities due to a pandemic was £58 billion [44].

Secondary hospital acquired infection after contracting influenza can contribute to further productivity loss due to time unable to work. Although not in the context of an influenza pandemic, Marchetti found that lost productivity due to for premature death of 45–64 year olds as £493,362 for a US population (using a currency conversion rate of $1 = £0.72).[58, 59] Lost wages due to an inability to work were calculated by multiplying the incremental length of stay by an age blended value of a lost workday. In a Belgian hospital, Vrijens et al found that the mean additional length of stay in hospital for patients who acquired secondary infections was 7.8 days (95% CI 5.1 – 10.5), and varied by the type of acquired infection, from 4.6-12 days.[60] This study costed the additional days from a healthcare perspective but did not account for the cost due to loss in productivity.[60]

Scenario 4: Catastrophic antimicrobial resistance (AMR)

In the underlying analyses by both KPMG and RAND for the O'Neill review, where the adverse scenarios of AMR levels increasing by 40% initially and remaining constant to 2050 (scenario C), and increasing to 100% initially and remaining at 100% (scenario D), the cumulative reductions in global GDP were estimated to be roughly £6 trillion (scenario C) and £10.2 trillion (scenario D) respectively, using a currency conversion rate of $1 = £0.72 [23].

The World Bank also proposed that by 2050, in a low AMR scenario, GDP would fall by 1.1%, while in a high AMR scenario, it would fall by 3.8% (corresponding to annual shortfalls of up to ~$1 trillion per 1% GDP) [61]. If 10% of these modelled costs were averted through AMR containment measures, high-income countries would still obtain benefits of £0.6 trillion and £1.9 trillion in the low and high AMR scenarios respectively [61] (using a currency conversion rate of $1 = £0.72). The AMR levels that corresponded to these low and high scenarios were not reported, and neither were any health impacts for the scenarios.

Lastly, the UK National Risk Register projected economic impacts of £100 million to £1 billion in the UK (among other mortality, societal and environmental impacts) [26].

Appendix 2 - Risk workshops with key opinion leaders

Workshop format and structure

Three workshops were held with different groups of key opinion leaders (KOLs), in order to harness expert opinion on each of the proposed adverse scenarios, and to obtain a collective assessment of the frequency and severity of impact of each scenario. The initial interdisciplinary workshop was attended by 9 KOLs with expertise in healthcare management, health economics, healthcare management, clinical care and microbiology. Subsequent health economics and clinical workshops were attended by 2 KOLs each. A semi-structured format was used, where the structure of pre-defined logic models for each scenario, specified in Figure A2.1, were followed. The key qualitative findings are summarised in this section, while the quantitative findings were combined with scoping review findings and are summarised within Appendix 3.

Figure A2.1: Logic model used in each scenario, in the initial risk workshop

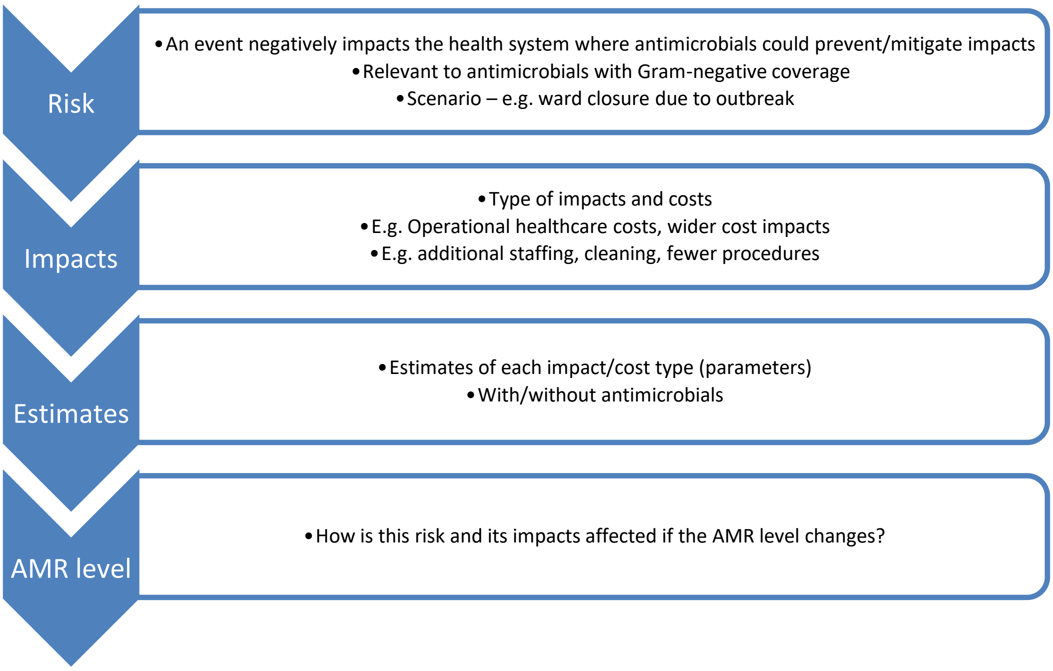

Key findings from workshop 1 (multidisciplinary)

The initial workshop concentrated on direct operational impact on the UK health service. The KOLs provided both numerical estimates of probabilities, scale of impacts (e.g. bed-days lost) and unit costs for the in-scope scenarios, and their qualitative assessments of the estimates and of the scenarios.

General comments

The KOLs stressed that the health service in the UK is deeply interconnected. Projects assessing the value of AMs should try to take a comprehensive view of all costs associated with patients' pathways throughout different parts of the healthcare system, and account for suboptimal levels of healthcare functioning even at normal times, e.g. due to understaffing. The project should consider which parameters are most important and are based on good quality evidence, as opposed to those that were not (e.g. in a heatmap).

Additionally, the health service is stretched. Risk mitigation and insurance value approaches could involve a new therapeutic, but could also involve having better reserves of nurses. Healthcare-related impacts are not only to patients but also to staff, as staff are sent home to protect patients and other staff. The flow of patients into and out of hospitals and through wards was crucial to estimating impacts– the lack of bed availability means that there are impacts both on those patient who should be entering and leaving.

Thirdly, the health service is regional. Infection rates vary by region and hospital, and the mixing of patients between locations may cause spread. Novel antimicrobial-related infection outbreaks tend to be more prominent in big cities, and the durations of these outbreaks are variable and may depend on hospital unit type and ethnicity.

A novel antimicrobial may additionally ameliorate AMR by enabling the infected patient to recover more quickly and get discharged from hospital earlier, thereby reducing the spread of infection. As patient mix in hospitals is heterogeneous, many

of them will be vulnerable to infections. Long term care facilities could be harbouring bacteria, but residents do not necessarily develop an infection that is relevant to a novel antimicrobial, hence it is not fully effective for treating all infections.

Scenario 1: Ward closure

Novel antimicrobials would be relevant to multiple resistant pathogens that could cause infection outbreaks triggering ward closures. Ward closure is equivalent to reducing admissions to the ward. The ward is normally still fully occupied at the start of its closure, so the number of lost bed days due to closure increases as outbreak progresses.

The scale of closures differs by location. ranging from 3-4 patients per week for whom a novel antimicrobial would be useful and one infection outbreak per year lasting several months, to several thousand patients being affected in a 3 month-long ward closure across UK regions.

Different wards have different characteristics. The proportion of single bed wards of all wards varies across hospitals.[62] As newer hospitals are more likely to be designed to have single bed wards, ward closures are likely to be less frequent. Intensive treatment units (ITU) have high network centrality and are amenable to disease transmission, however outbreaks are low as these units are monitored closely to prevent spreading of infections to other wards. In contrast, infection spreading between medical and surgical wards is rampant.

There may be 3000 lost bed days where a novel antimicrobial is relevant, costing the health system £300,000 and occurring once per year. An example of an extreme scenario is a norovirus closing the whole of a smaller hospital, and multiple ward closures across a big city.

In terms of the opportunity cost of bed occupancy, the impact varies by type of bed, as taking up acute beds has a different opportunity cost to taking up more suitable types of beds for the specific patients. Recent research estimated that the use of vaccines could avoid blockages of hospital beds due to vaccine-preventable outcomes, such that the opportunity costs avoided are twice the operational costs saved.[63] There would also be substantial additional costs from knock-on impacts of the closure (e.g. being assigned to an acute bed when not appropriate), with an exponential impact outside of the hospital. There could also be deaths on waiting list due to ward closures.

Scenario 2: Unavoidable shortage of conventional antimicrobials

The antimicrobial supply chain is delicate. Two shortages due to supply chain disruptions have happened within the last 5 years, even though they were expected to occur once in a lifetime.

The Tazocin shortage identified in the scoping review (Appendix 1) was also discussed by the KOLs, who highlighted that shortages could arise from a single manufacturing component having key person or organisation dependency. The Tazocin shortage lasted for approximately 1 year, but supply chains can be disrupted for more than 1 year.

There has been a worldwide shift in antimicrobial buying practices, causing inflation in costs of alternatives. Alternatives may be substantially more expensive even before price inflation, and are usually as effective as the original. During the COVID-19 pandemic, health providers could not buy common antimicrobials due to panic buying. A novel antimicrobial is likely to be a niche antimicrobial that is not toxic, so it is a more appealing option for treatment.

Scenario 3: Viral respiratory pandemics

The factors contributing to the impact on health systems include length of ventilation, aversion of death, earlier removal of mechanical ventilation or from ITU bed, the efficacy of a novel antimicrobial compared to other agents, ventilator days saved, and frequency of secondary infections. Regarding the frequency of secondary infections, a recent study that estimated 5-15% of admitted COVID-19 cases acquired secondary infections [22] was mentioned. In terms of the number of patients admitted to hospital due to viral respiratory illness during a pandemic, a study that reported 46,215 admissions in England during the 2017/18 influenza pandemic [64], and the number of admissions due to Covid-19 (400,385 as at 3 June 2021) [65] were mentioned. There was no data on the duration of initial treatment with antimicrobials that are not effective.

The mix of 20% of hospital patients in ward beds, 40% in High Dependency beds and 40% in Intensive Care beds result in a consolidated cost of £1,200 per bed when using the ward average values provided in the Welsh Government's delivery plan for the NHS in 2011/12 [66].

Humans are constantly increasing the ways of spreading infection, through new technology and social practices. Human activities that induce climate change are likely to have increased the risks of future pandemics, e.g. aggressive agriculture leads to more zoonotic diseases. Heavy winters due to flu hospitalisations occur roughly every 5 years. However in recent years, every winter has been a bad winter and the flu season is getting longer. This also has knock-on impact on staff, who have to take sick leave if they display symptoms, and health system productivity, as logistics are adversely affected and patients may not be assigned to the right beds.

The scale of infections depend on how often the flu vaccine is effective. Vaccine effectiveness varies from 30-70%. However, in the whole population and over time, effectiveness may be lower as newer strains of the same virus evolve over the flu season, and many people are not offered vaccine in the UK (younger adults with no respiratory disease).

The COVID-19 situation was a focal point for the discussion of this scenario. COVID-19 was previously regarded as a very rare event, however the pace of global pandemics has been increasing and now severe pandemics like COVID-19 could be expected every 10-15 years. In-hospital transmission for COVID-19 has been extremely high, and a high percentage of infections in a flu pandemic are expected to be Gram-negative. A novel antimicrobial is likely to only treat 2/3 of the worst flu-related pathogens.

Key findings from workshop 2 (health economics)

Societal impacts framework and estimation methods

Initially, the KOLs discussed whether the health system's risk appetites differed across these scenarios, which would affect how they prioritise risk mitigation or preventative measures for these scenarios. The first three scenarios of ward closures, unavoidable shortage of conventional antimicrobials and viral respiratory pandemics, appear to be risk neutral scenarios, which may plausibly occur over the next 10 years. In contrast, the catastrophic AMR scenario is more likely to be viewed as a risk averse scenario, as although the probability of its occurrence in the next 10 years may be low, should the event occur, the event would be extremely costly and the government might be willing to invest substantially to avoid this scenario. The difference in risk appetite between the first 3 scenarios and the catastrophic AMR scenario meant that deliberation around their insurance values should therefore be kept separate. The government's perspective is to be risk neutral for most scenarios, however COVID-19 may have prompted the government to look more at risk-averse scenarios, such as the catastrophic AMR scenario that was added to this analysis.

The proposed use of the NICE wider societal benefit modelling methodology [1] was endorsed by the KOLs. This methodology was published in 2013 as part of wider NICE project to develop its value-based care framework. The KOLs recommended using this approach without modification, although modifications would be allowable if explained or justified, particularly if they would result in a large deviation from the model results reported by NICE.

In terms of the types of wider societal benefits in scope, the paid productivity and informal care models in the NICE methodology were relevant and the most pertinent types of societal impact. The inclusion of unpaid productivity impacts was also recommended, as its inclusion would reduce discrimination due to differing work patterns, e.g. by sex, on a national level.

The NICE framework for estimating wider societal benefits follows just the human capital approach and does not consider friction costs, such as productivity impacts due to death.

Under normal circumstances, the KOLs advised that the use of the age, sex, quality of life (QoL) and ICD-10 hospital patient profile [50] identified in the scoping review (Appendix 1) was reasonable.

As QoLs are used to estimate societal benefits in the NICE framework, double counting the impact on QALYs may arise if incremental cost effectiveness analysis was carried out. There is literature to justify any view on this issue, e.g. using strategies that consider specific groups of patients might result in less double counting.

Scenarios 1-3: Ward closures, unavoidable shortage of conventional antimicrobials and viral respiratory pandemics

No specific considerations were discussed for the ward closure and shortage scenarios.

For the viral respiratory pandemic scenario, several types of costs additional to the operational healthcare costs, health impacts and societal impacts were suggested, such as the cost to NHS of enlarging the health system to deal with large outbreaks, and knock on impacts on education, infrastructure development, waiting lists and social care. The cost of healthcare staff sickness is normally accounted for in economic evaluations, while the knock on impact on those they care for is not.

The availability of anti-infectives in this scenario is an important consideration when estimating the number of patients affected. Additionally, if hospitals discharge patients early due to a pandemic, their shorter bed days may correspond to greater post-discharge care needs.

Scenario 4: Catastrophic AMR

The catastrophic AMR scenario might be similar to a viral respiratory pandemic in terms of its impact. Additionally, learnings from the COVID-19 pandemic could be a proxy for this scenario, particularly around the enablement impact of medical procedures, separate from the insurance value.

Key findings from workshop 3 (clinicians)

Scenario 1: Ward closure

Hospitals are likely to be running with close to 100% bed occupancy over the next 5 years, so small disruptions can have a large impact on the health system and patient outcomes.

The approach to translate bed days lost due to ward closure to the number of affected patients:

- Varies by type of ward – shortest for elective wards, 5-7 days per patient for a cardiac ward, more variable for a medical ward, potentially up to twice that for the cardiac ward.
- Differs between elective and emergency wards. Elective surgery is also most affected when a ward closes.

Therefore an average length of stay of 5-7 days per patient could be used. Alternatively, the duration of ward closure could be used as the denominator to derive number of affected patients from bed days lost. Ward closure durations ranging from 7 days to 6 months have occurred in London.

There is more heterogeneity in affected patients' length of stay due to ward closure: if patients in a closed ward are not infected, bed days may be reduced by 1 bed day as trying to get them out sooner, but if they are infected, they may remain in the closed ward for an additional 7-14 days. Less than 1% of patients trapped in the ward are likely to get an infection in a medical ward afterward, although in a surgical ward it could be 5%.

In terms of productivity and informal care duration, patients on a surgical waiting list could die whilst waiting on the list. Whether the person can wait safely at home is difficult to quantify. Most orthopaedic patients would be retired and will not contribute substantially to paid productivity.

In terms of affected patient profile, the specialties most impacted that would be relevant to a novel antimicrobial would be renal units, vascular units and cardiothoracic units. The greatest burden for closures would be in high acuity patient wards where there is a delay to their care, delay in their return to work, and the cost of death. ¾ of patients affected are likely to be in high acuity areas and ¼ in low acuity areas. In a hospital ward, of 90 colonised patients, ~5 were infected. The mean age of patients could be the 59 years reported by a study of hospitalised patients in UK [47]. The mean ages for admitted patients by type of admission (ICD-10 diagnosis code) are reported by HES [67].

Scenario 2: Unavoidable shortage of conventional antimicrobials

It can be assumed that no patients go without treatment in this scenario, if we also assume that the novel antimicrobial supply chain is not affected and the novel antimicrobial is always available. The 3000 affected patients estimated at the initial workshop is a reasonable estimate but the actual number varies with the type of antimicrobial is used. Similar to the ward closure scenario, the mean age could be an estimate of the national average.

To date, the KOLs have always had either a single alternative or multiple alternatives available. The issues have either been that the alternative is more expensive, or the alternative is normally priced but the manufacturer has profited. They have also had to use multiple alternatives as there was insufficient supply for a single alternative.

There is little evidence on whether the use of alternatives has any impact on patients, as there are few head-to-head trials. When prescribing an alternative, the route of administration may change, and patient allergies need to be considered.

There is also little evidence of the productivity and informal care impact on patients, although there could be a delay of 2-3 days in procuring the alternative, and obtaining approval and delivery. The additional cost to the health system rather than this delay would be the main impact.

Scenario 3: Viral respiratory pandemics

For the typical length of stay, among patients with viral pneumonitis, with secondary bacterial infections, for whom the novel antimicrobial would be relevant, the mean length of stay is probably going to be 14-21 days (minimum of 14 days if they were fit and healthy)

In terms of duration between discharge and recovery, patients are likely not to re-enter work for 2 months on average, 4-6 weeks minimum if healthy, 6 months at maximum. The distribution of the duration is such that less than 10% are expected to be at the lower end, 66% at the middle and long tail at the higher end. An extreme example is a patient who was in hospital for 157 days, then off work for a much longer duration after discharge. Return to work patterns will also depend on the type of work, e.g. the duration is longer if they are involved in physical labour. Informal care is likely to be required for the duration between discharge and recovery, and possibly even after recovery, if they suffer from permanent disability.

Those aged over 65 years are more likely to need informal care in this time, and some may never recover and will always need some form of care throughout their lives after discharge (i.e. the model could underrepresent informal care costs for this group of patients). Those much older are more likely to die soon after. The mean age of patients was likely to be higher than the national average, as affected patients tend to be aged 65 years and above.

In terms of subsequent infections for those who survive, less than 2% of these patients would have chronic long term problems characterised by recurrent infection that require expensive antimicrobial treatment.

For mortality profiles, a systematic review reported that survival in patients with limited treatment options ranged from 35-100% if given a novel antimicrobial (an average of 65% could be used) [68]. Whereas if a non-active agent e.g. Tazocin was used, or if there was no effective antimicrobial, survival could be as low as 0%. In a cohort study of treatment of carbapenemase-producing K. pneumoniae, survival beyond 30 days improved substantially with the use of combination antimicrobials as opposed to monotherapy [69].

Scenario 4: Catastrophic AMR

The probability of the UK reaching a catastrophic AMR scenario (as described by the O'Neill review and KPMG[23]) by 2040 was estimated to be 50% by one KOL, as the UK was perceived as similar to other countries that have had adverse AMR outbreaks (Italy, Israel, and many countries in South and East Asia), and there is nothing special that protects the UK. The MRSA outbreak in the 2000s in UK also demonstrates that the UK fares no better in preventing AMR outbreaks. However, the other estimate of 5% (with wide confidence intervals around this estimate) was based on the belief that UK is better positioned, good control in terms of agricultural use of AMs, and that the UK differs from southern Europe in terms of power in legislation for prescription-only medicines. Additionally, Extended Spectrum Beta-Lactamase rates that skyrocketed in the 1990s and early 2000s have plateaued off.

For the increase in AMR levels by 40% that were described in the catastrophic scenario, the 5% probability estimate is reasonable, and it is also reasonable to expect the infection rate to double in this scenario. This degree of AMR level and infection rate impact could be due to the ageing and increasingly comorbid population, although a gentle rather than sudden increase in AMR over time would be associated with this trend.

Novel antimicrobials could reduce the severity if the event occurred, but not reduce the chance of it happening.

Risk assessments for model parameterisation

Based on these quantitative responses during the workshop and through post-workshop follow up, the inputs (frequencies, unit costs, durations and patients affected) required for the synthesis of model parameters were summarised (Appendix 3), and the frequencies and severities of the impacts derived from these inputs used in the modelling of insurance value are listed in Appendix 4.

For the main analysis, AMR levels were assumed to remain at current levels, while for the increasing AMR situation, AMR levels were assumed to be at current AMR levels at year 0, then increasing linearly year-on-year to the projected high AMR levels at 2030, of 11.8% for E. coli, 15.0% for K. pneumoniae and 8.0% for P. aeruginosa (Appendix 3).

Appendix 3 – Model parameters based on findings from scoping review and workshops

Scenario 1: Ward closures

Model parameters	Assumption	Source	Comment
Reduction in bed days lost due to having an anti-microbial available (F)	20%	Workshop discussions with KOLs and refined with KOLs	The KOLs agreed that having an antimicrobial available would reduce length of stay. Further discussion with clinician KOL to quantify the benefit, who felt that length of stay would be reduced by ~30% but that not all of the benefit will be carried through so therefore to assume 20%.
Annual frequency of event described in England (F)<br>- Moderate case<br>- Severe case	1 event in 3 years (increased to 1 event in 1 year)<br>1 event in 10 years (increased to 1 event in 4 years)	Halaby et al<br>Workshop discussions with KOLs	Halaby et al indicates a frequency of 1 event in 3 years which is in line with the moderate scenario. The more severe event was discussed at the workshop and the participants felt a reasonable estimate as would be rare.<br>For higher AMR levels, increase in incidence due to the higher number of AMR bacteria causing infections.
Bed days lost (F)<br>- Moderate case<br>- Severe case	3,000<br>10,000	Workshop discussions with KOLs	The more severe scenario is extreme but represents when there are a large number of multiple ward closures.
Additional staff costs per bed day lost (D)	£175	Otter et al<br>Increased slightly based on workshop discussions with KOLs	Otter et al shows additional staff costs of €228,000 (€205,000 - €251,000) for 1,206 bed days lost. Using a €1 = £0.86 FX rate this indicated ~£160 (£145 - £180).<br>However, KOLs indicated these costs are likely to be higher in reality to allow for higher staff costs in more extreme events and so the higher estimate has been used.
Opportunity cost to health system per bed day lost (D)	£500	Otter et al<br>Increased slightly based on workshop discussions with KOLs	Otter et al shows opportunity cost of €349,000 and €244,000 (€77,000 - €512,000) totalling a cost of €593,000 (€426,000 - €861,000) for 1,206 bed days lost. Using a currency conversion rate of €1 = £0.86, this indicated ~£420 (£300 - £600).

			However, KOLs indicated these costs are likely to be higher in reality when accounting for social care costs as well and so the higher estimate has been used.
Additional cost for screening and other precautions per bed day lost (D)	£75	Otter et al<br><br>Increased slightly based on workshop discussions with KOLs	Otter et al shows screening costs of €94,000 for 1,206 bed days lost. Using a currency conversion rate of €1 = £0.86 this indicated ~£70.<br><br>However, KOLs advised that costs are likely to be higher when accounting or the need for faster screening in such an event, so they have been increased slightly.
Additional cost for anti-infectives per bed day lost (D)	£100 (increased to £200)	Otter et al<br><br>Refined with KOLs	Otter et al showed the costs to be ~£40 (€54,000 across 1,206 bed days). Following discussions with the KOLs this was increased to allow for the increase in price and additional therapeutic drug monitoring that is often required in such cases.
Cleaning costs per bed day lost (D)	£20	Otter et al<br><br>Workshop discussion with KOLs	Proposed by one of the KOLs following discussions. In keeping with Otter et al which estimated a cost of ~£30 per bed day lost.
No. of excess deaths among patients with infections (H)<br><br>- Moderate case<br><br>- Severe case	 <br>5<br><br>30<br><br>(increasing by 50% in both cases)	Wong et al<br><br>Refined with KOLs	Initial values of 17 (moderate) and 510 (severe) excess deaths were estimated from the Wong et al systematic review of ward closure studies, which found one several studies that reported mortality rates of 4-85% (used to estimate severe case). These rates were applied to KOL estimates of number of patients affected (429 for moderate and 600 for severe), based on bed days lost and length of stay.<br><br>These estimates were revised downwards to 5 (moderate) and 30 (severe) excess deaths based on KOL clinical experience of ward outbreaks.
No. additional infections (H)<br><br>- Moderate case<br><br>- Severe case	 <br>10<br><br>60<br><br>(increasing by 50% in both cases)	Workshop discussion with KOLs and refined with KOLs	KOLs advised a secondary infection rate in hospital wards of 1-5% at the workshop, and advised estimates of 10 (moderate) and 60 (severe) additional infections after the workshop, based on clinical experience.
Infection duration (H)<br><br>- Moderate case<br><br>- Severe case	 <br>10 days<br><br>42 days	Refined with KOLs	The original estimate of 10 days remained unchanged for the moderate case. Whereas 14 days was to 42 days for the severe case, as from their clinical experience, the infection of bone, organ space, or in-dwelling

			medical device would typically require 6 weeks of therapy with an effective AM such as the novel antimicrobial.
Median age at death of affected patients (H)<br>- Moderate case<br>- Severe case	75 years<br>70 years	Refined with KOLs	KOLs advised median ages at death of 75 (moderate) and 70 (severe) years, as even though ages of affected patients are hugely variable, multiple drug-resistant organisms also affect younger patients, and median ages in surgical wards or renal wards may be much lower.
Additional bed days per patient (S)<br>- Moderate case<br>- Severe case	10 days<br>42 days	Refined with KOLs	One KOL proposed to increase the additional bed days per patient, from original estimates of 7 and 14 days to 10 and 42 days for the moderate and severe case respectively. This matched their estimates for infection duration and was based on their clinical experience.
Number of patients who would have been admitted had the ward not closed (S)<br>- Moderate case<br>- Severe case	400<br>2800	Refined with KOLs	One KOL proposed to significantly increase the number of patients who would be affected by a ward closure in a severe case from 500 to 2800. The moderate case was also increased less so but still substantially from 250 to 400.
Proportion of bed days where productivity affected (S)<br>- Moderate case<br>- Severe case	130%<br>150%	Discussed with KOLs	The slightly larger impact took into account the additional time that a patient was in poor health and was unable to work but was not yet assigned to the appropriate ward. These proportions were discussed with the KOLs.
Proportion of bed days where informal care needed (S)<br>- Moderate case<br>- Severe case	20%<br>90%	Refined with KOLs	One KOL agreed with the suggestion that in a severe case additional informal care would be needed for 90% of bed days. However, in the moderate case the original suggested value of 40% was reduced to 20%.
ICD chapter casemix for affected patients (S)	N (genitourinary): 25%,<br>J (respiratory): 25%,<br>I (circulatory): 25%,<br>C (neoplasms): 25%	Refined with KOLs	Workshop discussions with clinician KOLs suggested that patients who would be most affected by a ward closure would be renal units, vascular units and cardiothoracic units. ICD composition was refined and agreed after the workshop to be split equally amongst chapters N, J, I and C.

No. of patients affected (S)<br>- Moderate case<br>- Severe case	 429<br>600	Workshop discussion and follow up discussion with KOLs	These numbers were based on KOLs' estimates of bed days lost (3,000 and 10,000 bed days for the moderate and severe cases respectively), and a length of stay of 5–7 days discussed at the clinicians' workshop. The intended logic is that the patients affected are those who could not be admitted due to ward closure. These patient numbers were discussed with the KOLs.

Model parameter types: risk event frequency (F), related to operational costs to health system (D), health-related (H), and related to societal impacts (S)
The text in blue relates to the alternative model parameters used in the case of higher AMR levels

Scenario 2: Unavoidable shortage of conventional antimicrobials

Model parameters	Assumption	Source	Comment
Number of patients receiving treatment of the anti-microbial whose supply chain is disrupted across England per day (F)	3,000 (increased 4-fold)	PHE Surveillance Programme for Antimicrobial Utilisation and Resistance	Carbapenem usage was 0.052 per 1,000 inhabitants per day in 2019. Assuming the population of England is 55.98 million this corresponded to ~3,000 patients per day.
Proportion of shortages where the novel antimicrobial would be a suitable replacement (F)	25% (increased 4-fold)	Workshop discussions with KOLs, refined with further discussions with KOLs	Discussions with KOLs confirmed approximately 30% would be suitable for Assuming that the novel antimicrobial would be suitable for extended spectrum beta-lactamases (ESBLs) in the event of a carbapenem supply chain failure. KOLs confirmed that ESBLs represent ~15-25% of all Gram-negative bacteremia and as such 25% is a reasonable estimate.
Duration of shortage (F)<br>- Moderate case<br>- Severe case	 90 days<br>180 days	Barber et al and Gross et al<br>Workshop discussion with KOLs and refined with KOLs	Barber et al referenced a quarter-long Tazocin shortage, Gross et al referenced a half-year Tazocin shortage.<br>Discussion with the KOLs confirmed that longer shortages of other antimicrobials can also cascade through all others leading to shortages for 1-3 months.
Annual frequency of event described in England (F)<br>- Moderate case<br>- Severe case	3 events in 10 years (increased to 1 event in 1 year)<br>1 event in 10 years (increased to 1 event in 4 years)	Workshop discussion with KOLs for both scenarios	Based on clinical experience.<br>For higher AMR levels, increase in incidence due to the higher number of AMR bacteria causing infections.
Increase in daily cost of treatment (D)	£200 (additional cost of £200 for anti-infectives)	Workshop discussions with KOLs	Discussed that price increases can be very extreme depending on the availability of other alternatives and the geo-political situation. It was assumed that the cost would increase further due to higher AMR levels.
No. of excess deaths among patients with infections (H)<br>- Moderate case<br>- Severe case	0 (increased to 35)<br>0 (increased to 60)	Refined with KOLs	KOLs advised that currently, no deaths would be expected as multiple drugs are available. If there was a significant increase in AMR then using the estimate of ~3000 patients a day affected by a supply chain impact, then estimated excess deaths could increase to 35 (moderate) and 60 (severe).

No. additional infections (H)<br>- Moderate case<br>- Severe case	0<br>120 (increasing by 100% in the severe case)	Workshop discussion with KOLs and refined with KOLs	KOLs advised that currently, no additional infections would be expected in the moderate case. In the severe case, onward infections could be assumed to arise due to the difficulty in treating these infections.
Time to source alternative treatment (H)<br>- Moderate case<br>- Severe case	0 days<br>2 days<br>(decreasing to 0 in both cases)	Workshop discussion with KOLs and refined with KOLs	KOLs advised that there is unlikely to be a substantial delay in sourcing alternatives, and even if they needed to be shipped in from continental Europe, it should only take maximum of 48 hours., the time to source an alternative would decrease to 0, as hospitals would be forced to carry all AM agents as stock.
Median age at death of affected patients (H)<br>- Moderate case<br>- Severe case	75 years<br>65 years	Refined with KOLs	KOLs advised that shortages would disproportionately affect older people, so median ages for this scenario are older than those for the ward closure scenario.
Additional bed days per patient due to treatment delay (S)<br>- Moderate case<br>- Severe case	0 bed days<br>3 bed days<br>(decreasing to 0 in both cases)	Workshop discussions with KOLs	KOLs agreed that in the moderate case an alternative drug could be sourced with no implications for the patient and thus no additional bed days In a severe case there could be minor delays (up to 3 days) with procuring the alternative drug, therefore adding up to 3 bed days., the time to source an alternative would decrease to 0, as hospitals would be forced to carry all AM agents as stock.
Proportion of bed days where productivity affected, due to treatment delay (S)<br>- Moderate case<br>- Severe case	100%<br>100%	Refined with KOLs	KOLs advised that a proportional impact of the disrupted bed days on productivity for both the moderate and severe cases was reasonable.
Informal care required as a proportion of bed days per patient, due to treatment delay (S)<br>- Moderate case<br>- Severe case	40%<br>30%	Refined with KOLs	One KOL suggested a greater percentage of days between discharge and return to work would require informal care in the moderate scenario when compared to the severe scenario. This is due to more severe shortages on affecting a wider age range of patients (on general and surgical wards) rather than mainly elderly patients. The reduced median age will lead to less proportionate informal care. However, given the greater number of bed days in the severe scenario, this still translates into a greater number

			of absolute days requiring informal care in the severe scenario, as would be expected.
ICD chapter casemix for affected patients, due to treatment delay (S)	N (genitourinary): 25%,<br><br>A (other infectious/parasitic): 50%,<br><br>J (respiratory): 25%	Refined with KOLs	KOLs agreed that the main ICD chapters covered would be for those that cover key relevant indications for the novel antimicrobial (N, A and J). KOLs refined the proportions to be 50% for ICD chapter A, as there are additional complications that fall under this chapter for which the novel antimicrobial may be used.

Model parameter types: risk event frequency (F), related to operational costs to health system (D), health-related (H), and related to societal impacts (S)
The text in blue relates to the alternative model parameters used in the case of higher AMR levels

Scenario 3: Viral respiratory pandemics

Model parameters	Assumption	Source	Comment
Proportion of hospitalised patients who have secondary bacterial infection (F)	10%	Rawson et al<br><br>Workshop discussion and further discussion with KOLs	Rawson et al reported that 8% of patients with COVID-19 had a secondary infection and 11% of those without COVID-19. Overall this was ~10%.
Proportion of hospitalised patients with secondary bacterial infections requiring the novel antimicrobial (F)	8% (increased to 12%)	Rawson et al<br><br>Refined with KOLs	Rawson et al estimated that ~9% of co-infected COVID-19 patients had a K. pneumoniae, E. coli or P. aeruginosa infection. (Summing those with such infections divided by the total number of COVID-19 co-infected patients). Following discussion with clinician KOL 8% has been used to allow for any differences between COVID-19 and non-COVID-19 patients.<br><br>For higher AMR levels, the situation may result in greater use of broad spectrum antimicrobials.
Number of patients admitted to hospital due to viral respiratory illness during the pandemic (F)<br><br>- Moderate case<br><br>- Severe case	50,000<br><br>400,000	Moss et al, UK Government Coronavirus dashboard<br><br>Workshop discussions	Moss et al found ~46,000 admissions within England during the 2017/18 influenza pandemic. Rounded to 50,000 to allow for changes over time to 2020/21.<br><br>On 3 June 2021 400,348 patients had been admitted to hospital in England as a result of coronavirus.
Annual frequency of event described in the England (F)<br><br>- Moderate case<br><br>- Severe case	1 events in 5 years<br><br>1 event in 20 years	Workshop discussion	These estimates were based on KOLs' clinical experience.
Cost per case requiring broad spectrum anti-microbials (D)	£1,000	Workshop discussions, consolidating costs published by Welsh Government	Assuming 20% of patients are in ward beds, 40% in High Dependency beds and 40% in Intensive Care beds (based on KOL clinical experience), result in a consolidated cost of £1,200 per bed when using the values provided in the Welsh Government's paper (averages of £413, £857 and £1932 per night for each respective ward type, in 2011/12). This has been rounded to £1,000.
No. of excess deaths among patients with secondary infections (H)	800	Rawson et al	Rawson et al reported that 8% of patients with COVID-19 had a secondary infection. KOLs advised that excess deaths during a moderate flu season are ~10,000 (to inform the moderate case), while there have

- Moderate case<br>- Severe case	12,000	Workshop discussion with KOLs and refined with KOLs	been up to 150,000 deaths from COVID (to inform the severe case). The estimated proportion of 8% was applied to these excess death estimates to obtain the excess death estimates for the subset of patients who had a secondary infection.
No. additional infections (H)<br>- Moderate case<br>- Severe case	 7,500<br>25,000	NHS COVID-19 Hospital Activity Statistics, Rawson et al<br>Workshop discussion with KOLs and refined with KOLs	KOLs advised using COVID-19 as a benchmark for a severe case and referring to COVID-19 hospital activity statistics reported by the NHS. The NHS reported that approximately 331,000 patients were admitted or diagnosed with COVID-19 between October 2020 and October 2021. Since Rawson et al reported that 8% of those with COVID-19 have secondary infections, Combining these two figures, the resulting estimate of roughly 26.5k additional secondary infections due to COVID-19 was rounded down to 25,000 for the severe case. KOLs also advised that there would be approximately 7,500 additional infections for the moderate case, based on their clinical experience.
Infection duration (H)<br>- Moderate case<br>- Severe case	 7 days<br>14 days	Refined with KOLs	KOLs advised that 7 and 14 additional bed days for the moderate and severe case respectively were suitable, due to a prolonged length of stay during a pandemic as the health system would be overloaded and less efficient.
Median age at death of affected patients (H)<br>- Moderate case<br>- Severe case	 82 years<br>82 years	ONS Average age at death COVID-19 user requested data<br>Informed by KOLs	KOLs advised using COVID-19 as a benchmark for a severe case and referring to national statistics on COVID-19-related deaths. The ONS reported median ages at death for those who died with COVID-19 in the fourth quarter of 2020 as 82 years, which was only slightly lower than the median age of 83.6 years in 2019 (described in the table for non-scenario specific assumptions below), hence the same median age was also used in the moderate case.
Additional bed days per patient (S)<br>- Moderate case<br>- Severe case	 7 bed days<br>14 bed days	Refined with KOLs	These estimates were aligned with those used for the infection duration (see above).
Proportion of bed days where productivity affected (S)<br>- Moderate case<br>- Severe case	100%<br>100%	Discussed with KOLs	KOLs did not expect a disproportionate impact on productivity for this scenario.

Duration between discharge and return to work (S)<br>- Moderate case<br>- Severe case	30 days<br>183 days	Workshop discussion with KOLs	KOLs agreed with the suggestion that the duration between discharge and return to work could be between 1 month and 6 months.
Percentage of days after discharge and return to work where informal care is required (S)<br>- Moderate case<br>- Severe case	80%<br>20%	Refined with KOLs	KOLs suggested a greater percentage of days between discharge and return to work would require informal care in the moderate compared to the severe case. This is due to a respiratory pandemic affecting all age groups (as opposed to only older people). However, given the greater number of days until return to work in the severe case, this still translates into a greater number of absolute days requiring informal care in the severe case.
ICD chapter casemix for affected patients (S)	J (respiratory): 100%	Workshop discussions with KOLs	KOLs agreed that given this scenario is a viral respiratory pandemic, all patients concerned will be under ICD chapter J (respiratory).

Model parameter types: risk event frequency (F), related to operational costs to health system (D), health-related (H), and related to societal impacts (S)
The text in blue relates to the alternative model parameters used in the case of higher AMR levels

Scenario 4: Catastrophic AMR scenario

Model parameters	Assumption	Source	Comment
Probability of occurrence by 2040 (F)	5-50%, with midpoint of 25% used as best estimate	UK National Risk Register<br>Workshop discussion	The UK National Risk Register projected that an AMR event would have between a 1 in 5-year to 1 in 100-year likelihood of occurrence. KOLs agreed with assigning the corresponding range of probabilities of 5-50% to a time horizon of 2021 to 2040.<br>The probability that the event would occur in 2021 to 2030 would be half of the probability that would occur by 2040, assuming a uniform probability of occurrence over time.
Health and cost parameters in the Pfizer HTA submission dynamic transmission model (D,H)	(Numerous values)	Gordon et al and Pfizer HTA submission	All health and cost parameters matched those in the Pfizer HTA submission, except AMR levels and bacterial infection rates under a catastrophic AMR scenario.
AMR levels for 2021 to 2050 (H)	Increase sharply by 40% and remain constant	O'Neill review and KPMG report	O'Neill review outlined a main adverse scenario where absolute AMR levels sharply increased by 40% while infection rates for 3 kinds of bacteria (3GCR E. coli and K. pneumoniae and methicillin-resistant S.

		Workshop discussion and refined with KOLs	aureus (MRSA)) sharply doubled, then remained stable until 2050. KOLs agreed that this scenario would constitute a catastrophic AMR scenario. KOLs also suggested a lower bound 20%, 25% and 10% and an upper bound of 40%, 50% and 30% for the 3 relevant pathogens to the novel antimicrobial that were modelled, E. coli K. pneumoniae and P. aeruginosa, respectively. These estimates were based on their clinical experience.
Bacterial infection rates (for 3GCR E. coli and K. pneumoniae and methicillin-resistant S. aureus (MRSA)) for 2021 to 2050 (H)	Sharply double and remain constant	O'Neill review and KPMG report<br>Workshop discussion and refined with KOLs	The projection of infection rates sharply doubling and remaining constant thereafter accompanied the O'Neill review main adverse scenario for AMR levels above. KOLs also suggested that infection rates increasing by 1.5 times and tripling would form a lower bound and an upper bound respectively.
Additional treatment days (S)	28 days	Refined with KOLs	One KOL suggested that the lower bound of additional treatment days to be 14 and the upper bound to be 42 days. The mean of these 2 values is 28 additional treatment days.
Proportion of bed days where productivity affected (S)	225%	Discussed with KOLs	The proportion was hypothesised to range between 200-250% of bed days, to reflect the disproportionately larger impact that a catastrophic AMR scenario could have on the economy in comparison to the number receiving treatment, due to an overwhelmed health system.
Subsequent informal care required as a proportion of hospital bed days (S)	60%	Workshop discussions and refined with KOLs	KOLs suggested subsequent informal care required as a proportion of hospital bed days would be 60% rather than 100% initially suggested. This was based on informal care disproportionately affecting the elderly, however, a catastrophic AMR event, would proportionality affect more young people, who require comparatively less informal care, thus reducing the percentage.
Proportion of patients with relevant infections where the novel antimicrobial would be a suitable replacement (S)	80%	Refined with KOLs	KOLs agreed with the 80% proportion.

Model parameter types: risk event frequency (F), related to operational costs to health system (D), health-related (H), and related to societal impacts (S)
The text in blue relates to the alternative model parameters used in the case of higher AMR levels

Non-scenario specific assumptions

Model parameters	Assumption	Source	Comment
Prevalence of anti-microbial resistance in 2030 (D, H) to:<br>- E.coli<br>- K. Pneumoniae<br>- P. aeruginosa	11.8%<br>15.0%<br>8.0%	Alvarez-Uria et al and Cravo Oliveira Hashiguchi et al<br>Refined following discussions with KOLs	Projected AMR prevalences for carbapenem resistant bacteria reported by Alvarez-Uria et al (E.coli and K. Pneumoniae) and Cravo Oliveira Hashiguchi et al (P. aeruginosa) were used. For K. Pneumoniae, KOLs advised that the reported AMR level of 52% is extraordinarily unlikely due to prescribing legislation within the UK. A lower estimate of 15.0% was recommended, to be more in line with the other bacteria and to be more UK relevant.<br>These parameters were not directly used in the models, but indirectly used to inform other model parameters and characterise the scenarios.
Infection duration in absence of scenario (H)	14 days	Workshop discussion and refined with KOLs	KOLs advised that the average length of stay in hospital if a patient is infected is 7-42 days, and 14 days on average.
Median age at infection in absence of scenario (H)	65 years	NHS Digital Hospital Admitted Patient Care Activity, used in Pfizer HTA submission<br>Consolidated value agreed with KOLs	NHS Digital Hospital Admitted Patient Care Activity reported average ages of patients admitted with complex intra-abdominal infections, complex urinary tract infections and hospital/ventilator-acquired pneumonia (cIAI, cUTI and HAP/VAP) of 53, 73 and 66 years respectively, in 2018/2019. KOLs advised a best estimate of 65 years, a lower bound of 50 years and an upper bound of 70 years.
Median age at death in absence of scenario (H)	83.6 years	ONS: Most common age at death	ONS reported that the median age at death in England and Wales in 2019 was 83.6 years.
No. relevant infections in absence of scenario (H)	800,000	NHS Digital Hospital Admitted Patient Care Activity	NHS Digital Hospital Admitted Patient Care Activity reported that 793,929 patients in England were admitted with any of cIAI, cUTI and HAP/VAP, in 2018/2019. This was rounded up to an estimate of 800,000 relevant infections.
Quality of life (QoL)<br>- While infected with pathogen of interest<br>- While uninfected (H)	0.6<br>0.8	Review of clinical studies in Pfizer HTA submission<br>Consolidated value agreed with KOLs	QoL values consolidated from QoLs for infected/uninfected patients for the relevant indications of cIAI, cUTI and HAP/VAP respectively: .0.60/0.85, 0.68/0.78 and 0.58/0.78. These values were selected through a literature review of clinical studies conducted for the Pfizer HTA submission. The consolidated and rounded values of 0.6/0.8 were agreed with KOLs.

Value of a statistical life (VSL) (H)	£30,000 per QALY	NICE guidance, HM Treasury Green Book	The VSL per QALY was taken to be the the NICE willingness to pay threshold of £30,000. The HM Treasury Green Book VSL of £60,000 per life year lost and per QALY was also relevant, and was used as a parameter in sensitivity analysis
Median patient age (S)	59 years	Rowen et al (EEPRU)	The Rowen et al (EEPRU) report used an average age of 59 years that was summarised from a study of inpatients in the UK.
Sex profile of patients (S)	50.8% men and 49.2% women	Rowen et al (EEPRU)	The Rowen et al (EEPRU) report used a sex profile with 49.2% women that was summarised from a study of inpatients in the UK.
ICD chapter patient casemix (S)	Detailed list of proportions of patients and their average QoL per ICD chapter	Soares et al	Soares et al estimated a detailed casemix from a study of patients in a UK hospital. KOLs advised that the source of the casemix is suitable.
Average wages in the UK (S)<br>- Net hourly wage of employees<br>- Gross weekly wage	 £11.55<br>£576	ONS: Average weekly earnings and HM Government income tax calculator	The value for gross weekly wage is from the ONS report of average weekly earnings in Great Britain at June 2021. Net weekly wages were calculated from gross weekly wages by the HM Government income tax calculator, then converted into an hourly wage assuming a 40-hour work week.

KOL: Key Opinion Leader
Model parameter types: risk event frequency (F), related to operational costs to health system (D), health-related (H), and related to societal impacts (S)
The text in blue relates to the alternative model parameters used in the case of higher AMR levels

Appendix 4 – Estimating the frequency and severity of risk events for each scenario

For each scenario, frequency estimates for risk events considered to be moderate and for those considered to be severe were obtained and refined through the scoping review, risk workshops and KOL advice. These frequencies are summarised in Table A2.

Severity impacts for each scenario and perspective were calculated from model parameters for the moderate case and severe case obtained via the same process. For the catastrophic AMR scenario, operational healthcare cost and health impacts were not estimated using this formula and instead estimated using a dynamic transmission model [70]). Otherwise, severity impacts were estimated using the formulae in the subsections below and are also summarised in Table A2.

Operational healthcare cost

For the ward closure scenario:

Unit costs were the sum of:

- Additional staff costs per bed day lost
- Opportunity cost per bed day lost
- Additional cost for screening and other precautions per bed day lost
- Additional cost for anti-infectives per bed day lost
- Cleaning costs per bed day lost

Operational cost = Unit costs × Proportion of cases where the novel antimicrobial would be relevant × Bed days lost

For the unavoidable shortage of conventional antimicrobials scenario:

Operational cost = Increase in daily cost of treatment × Number of patients receiving treatment of the anti-microbial whose supply chain is disrupted across England per day × Proportion of disruptions where the novel antimicrobial would be a suitable replacement × Duration of shortage

For the viral respiratory pandemic scenario:

Operational cost = Proportion of patients who have secondary bacterial infection × Proportion of these patients requiring the novel antimicrobial × Cost per case requiring broad spectrum anti-microbials × Number of patients admitted to hospital due to viral respiratory illness

Health impact (in monetary terms)

Health impact = QALYs lost × NICE Value per QALY (Main analysis)

or Max(QALYs lost, years of life lost) x HM Treasury Value of a statistical life (Sensitivity analysis)

QALYs lost and years of life lost were estimated using standard health economic methods from the following inputs: number of patients whose health was affected, quality of life while infected and uninfected, median ages at infection and death, and differences in time spent infected and uninfected.

Key aspects of societal impact: productivity and informal care impacts

Productivity impact = Sum of paid and unpaid productivity loss per person day × Proportion of cases where the novel antimicrobial would be relevant × Bed/treatment days lost × Proportion of bed/treatment days where productivity affected

Informal care impact = Informal care cost per person day × Proportion of cases where the novel antimicrobial would be relevant x Bed/treatment days lost × Proportion of bed/treatment days where informal care needed

The unit costs of paid productivity loss and unpaid productivity loss and informal care cost per person day were estimated using the models recommended by NICE [1]. The aggregate societal impact estimated in this study was the sum of the values for productivity impact and informal care impact. These values are in terms of bed days for the ward closure and viral respiratory pandemic scenarios, and in terms of treatment days for the unavoidable shortage of conventional antimicrobials and catastrophic AMR scenarios. For the informal care impact of the catastrophic AMR scenario, ‘Proportion of bed/treatment

days where informal care needed' was substituted with 'Subsequent informal care required as a proportion of hospital bed days'.

Table A2: Summary of frequency and severity of impacts for each of the first three scenarios, to input into the insurance value model

(a) Main analysis (AMR at current levels)

Impact size	Scenario names	Frequency of moderate-sized event	Operational healthcare cost	Operational healthcare cost + health impact	Operational healthcare cost + health + productivity and informal care impact
Moderate impact	Ward closure	1 in 3 years	£522,000	£693,923	£768,396
	Unavoidable shortage of conventional antimicrobials	3 in 10 years	£13,500,000	£13,500,000	£13,500,000
	Viral respiratory pandemic	1 in 5 years	£400,000	£7,526,833	£7,813,451
Severe impact	Ward closure	1 in 10 years	£1,740,000	£3,381,530	£3,682,396
	Unavoidable shortage of conventional antimicrobials	1 in 10 years	£27,000,000	£27,010,213	£29,805,364
	Viral respiratory pandemic	1 in 20 years	£3,200,000	£148,060,625	£152,359,405

(b) Projected high AMR levels at 2030

Impact size	Scenario names	Frequency of moderate-sized event	Operational healthcare cost	Operational healthcare cost + health impact	Operational healthcare cost + health + productivity and informal care impact
Moderate impact	Ward closure	1 in 1 year	£582,000	£839,909	£914,382
	Unavoidable shortage of conventional antimicrobials	3 in 10 years	£63,000,000	£63,000,000	£63,000,000
	Viral respiratory pandemic	1 in 5 years	£600,000	£11,090,250	£11,520,177
Severe impact	Ward closure	1 in 4 years	£1,940,000	£3,581,530	£3,882,396
	Unavoidable shortage of conventional antimicrobials	1 in 10 years	£126,000,000	£131,612,873	£131,612,873
	Viral respiratory pandemic	1 in 40 years	£4,800,000	£220,490,938	£226,939,108

Notes:
For the main analysis, AMR was assumed to remain at current levels over the 10 year horizon ((a) throughout), while for the increasing AMR levels case, AMR was assumed to increase from current levels to the high AMR levels projected for 2030 (interpolating between (a) and (b)).
The insurance value for the fourth scenario, catastrophic AMR, was analysed using a different model that did not require model inputs in this format.

Table A3: Distributional parameters for each of the first three scenarios, to input into the Monte Carlo simulation model for insurance values (for the joint Poisson-Generalised Pareto distributions)

(a) Main analysis (AMR at current levels)

**Parameter type**	**Scenario names**	**Operational healthcare cost**	**Operational healthcare cost + health impact**	**Operational healthcare cost + health + productivity and informal care impact**
Frequency parameter for Poisson distribution	Ward closure			0.6667
	Unavoidable shortage of conventional antimicrobials			0.8000
	Viral respiratory pandemic			0.2750
Scale (beta) parameter for Generalised Pareto distribution	Ward closure	675,761	754,229	830,577
	Unavoidable shortage of conventional antimicrobials	13,340,822	13,343,554	13,280,395
	Viral respiratory pandemic	1,244,601	20,796,307	21,610,778
Shape (xi) parameter for Generalised Pareto distribution	Ward closure	0.3072	0.8000	0.7965
	Unavoidable shortage of conventional antimicrobials	0.0010	0.0010	0.0725
	Viral respiratory pandemic	0.0575	0.7873	0.7810

(b) Projected high AMR levels at 2030

**Parameter type**	**Scenario names**	**Operational healthcare cost**	**Operational healthcare cost + health impact**	**Operational healthcare cost + health + productivity and informal care impact**
Range of frequency parameters for Poisson distribution	Ward closure			0.7333-1.9333
	Unavoidable shortage of conventional antimicrobials			0.8-0.8
	Viral respiratory pandemic			0.275-0.275
Range of scale (beta) parameters for Generalised Pareto distribution	Ward closure	685269-698925	762980-891289	850287-972981
	Unavoidable shortage of conventional antimicrobials	15786638-52447129	15864047-53654597	15883731-53702374
	Viral respiratory pandemic	1807567-2280872	21286636-26405086	22121470-27458166
Range of shape (xi) parameters for Generalised Pareto distribution	Ward closure	0.1496-0.2842	0.4692-0.779	0.4635-0.7594
	Unavoidable shortage of conventional antimicrobials	0.001-0.001	0.001-0.001	0.001-0.0506
	Viral respiratory pandemic	0.1462-0.1462	0.7879-0.7947	0.7815-0.7881

Notes:
For the main analysis, AMR was assumed to remain at current levels over the 10 year horizon ((a) throughout), while for the increasing AMR levels case, AMR was assumed to increase from current levels to the high AMR levels projected for 2030 (interpolating between (a) and (b)).
Therefore, the scale and shape parameters reported for (a) relate to single-year severity estimates prior to inflation and discounting over the 10-year projection period, while the range of parameters reported for (b) reflect the increasing trend in the inflated and discounted single-year severity estimates from the 1st to the 10th projected year.
The insurance value for the fourth scenario, catastrophic AMR, was analysed using a different model that did not require model inputs in this format.

Sensitivity analyses

For sensitivity analyses, the frequencies at the lower and upper ends of the range specified in the risk assessment (Appendix 3) were used. For severity parameters, the high estimates specified in the risk assessment for: opportunity cost per bed, proportion of hospitalised patients with secondary bacterial infections requiring the novel antimicrobial, infection duration, age at infection (for scenarios other than catastrophic AMR), AMR levels and infection rates (for the catastrophic AMR scenario) and value of life were used; and bed days lost during ward outbreak at the low end of the range was also used. Sensitivity analysis

using an alternative discount rate of 1.5% was also conducted, as this lower rate was recently considered for recommendation by NICE and the Treasury [34, 35]. Sensitivity analysis using alternative inflation rates of 1% and 5% were also conducted, although inflation rate is not a key parameter in economic evaluations in the UK.

Appendix 5 – R code for Monte Carlo simulation

```
# Appendix - R code for Monte Carlo simulation, using a joint Poisson (frequency) and
Generalised Pareto (severity) distribution of insurance values
# Last edited: 24 March 2023

# Model settings
#install.packages("evir") # Load evir package for Generalised Pareto distribution
library(evir)
library(matrixStats) # Load matrixStats package to get quantiles by row/column

seedno<-5 # set the seed for generating pseudo-random numbers
nsim<-2*10^6 # set number of simulations to run
pctiles<-seq(0.001,0.999,by=0.001)  # setup vector of percentiles from 0 to 100%, increasing
by 0.1%

# Input parameters (specimen parameters and scenarios used here)
indata<-data.frame(Scenario_impact = c("Ward closures - Operational", "Ward closures -
Operational+health"), # Scenario name, then insurance value perspective, separated by a " - "
            Sens_type = c("Base case", "Base case"), # Sensitivity analysis name
            Year = c(0, 0), # Projection year
            Freq = c(0.667, 0.667), # Frequency parameter for the Poisson distribution
            Scale_beta = c(675700, 754230), # Scale (beta) parameter for the Generalised
Pareto distribution
            Shape_xi = c(0.307, 0.800)) # Shape (xi) parameter for the Generalised Pareto
distribution

# Setup output dataset formats
outdata<-indata[,c("Scenario_impact", "Sens_type", "Year")]
outmat<-matrix(rep(NA,3*nrow(indata)),ncol=3)
colnames(outmat)<-c("mean","percentile_90th","analytic_mean")
outdata<-cbind(outdata,outmat)

outheading<-paste(outdata$Scenario_impact,outdata$Sens_type,outdata$Year)
outsimdata<-matrix(rep(NA,length(outheading)*length(pctiles)),ncol=length(outheading))
colnames(outsimdata)<-outheading
outrawsimdata<-matrix(rep(NA,length(outheading)*nsim),ncol=length(outheading))

###########
# Monte Carlo simulation procedure

# loop Monte Carlo simulation across scenarios/settings/years
for (i1 in 1:nrow(indata)){
  # get model inputs
  lambda1<-indata$Freq[i1]
  beta1<-indata$Scale_beta[i1]
  xi1<-indata$Shape_xi[i1]
  analytic_mean<-lambda1*beta1/(1-xi1) # analytical mean of Generalised Pareto distribution is
[frequency * scale/(1- shape)]

  # insert lambda and simulate values from the Poisson distribution
  freq_sim<-rpois(n=nsim,lambda=lambda1)

  # setup output dataframe to hold simulated (and aggregated) prob-weighted severities
  joint_sim<-rep(0,length(freq_sim))

  # simulate severity 'freq_sim' times
  for (i2 in 1:length(freq_sim)){
    freq_n<-freq_sim[i2]
    if (freq_n==0){} else{
    # simulate severity using scale and shape parameters for the Generalised Pareto
distribution
    sev_sim_n<-rgpd(n=freq_n, mu =0, beta = beta1, xi = xi1)
    joint_sim[i2]<-sum(sev_sim_n)
    }
  }
  # get percentiles and mean
  outrawsimdata[,i1]<-joint_sim
  pctile_out<-quantile(joint_sim, pctiles)
  outsimdata[,i1]<-pctile_out
  outdata$mean[i1]<-mean(joint_sim)
  outdata$percentile_90th[i1]<-quantile(joint_sim, 0.9)
  outdata$analytic_mean[i1]<-analytic_mean
}

###########
# Summarising and aggregating simulation output

# get percentiles and mean from aggregated impacts (across years)
agg_outheading<-paste(outdata$Scenario_impact,outdata$Sens_type)
colnames(outrawsimdata)<-agg_outheading
agg_simdata<-t(rowsum(t(outrawsimdata), group = colnames(outrawsimdata), na.rm = F))
```

```
agg_pctile_out<-t(colQuantiles(x=agg_simdata, probs=pctiles))

# create output dataframe for mean and 90th percentile of aggregated simulations
agg_outdata<-matrix(rep(NA,2*ncol(agg_simdata)),ncol=2)
colnames(agg_outdata)<-c("mean","percentile_90th")
rownames(agg_outdata)<-colnames(agg_simdata)
agg_outdata[,"mean"]<-colMeans(agg_simdata)
agg_outdata[,"percentile_90th"]<-colQuantiles(x=agg_simdata, probs=0.9)

# further aggregate across scenarios (required for results for any xth percentiles)
agg2_outheading<-unlist(strsplit(agg_outheading, "- ", fixed=TRUE))
agg2_outheading<-agg2_outheading[c(FALSE,TRUE)]
colnames(outrawsimdata)<-agg2_outheading
agg2_simdata<-t(rowsum(t(outrawsimdata), group = colnames(outrawsimdata), na.rm = F))
agg2_pctile_out<-t(colQuantiles(x=agg2_simdata, probs=pctiles))

# create output dataframe for mean and 90th percentile of doubly aggregated simulations
agg2_outdata<-matrix(rep(NA,2*ncol(agg2_simdata)),ncol=2)
colnames(agg2_outdata)<-c("mean","percentile_90th")
rownames(agg2_outdata)<-colnames(agg2_simdata)
agg2_outdata[,"mean"]<-colMeans(agg2_simdata)
agg2_outdata[,"percentile_90th"]<-colQuantiles(x=agg2_simdata, probs=0.9)

# Summary results of insurance value (mean and 90th percentile) for each insurance value
perspective, projection year and type of sensitivity analysis
summ_insurance_value<-agg2_outdata
```

References

1. National Institute for Health and Care Excellence. Department of Health Documentation for wider societal benefits2013.
2. Wong H, Eso K, Ip A, Jones J, Kwon Y, Powelson S, et al. Use of ward closure to control outbreaks among hospitalized patients in acute care settings: a systematic review. Syst Rev. 2015 Nov 7;4:152.
3. Hansen S, Stamm-Balderjahn S, Zuschneid I, Behnke M, Rüden H, Vonberg RP, et al. Closure of medical departments during nosocomial outbreaks: data from a systematic analysis of the literature. Journal of Hospital Infection. 2007 2007/04/01/;65(4):348-53.
4. Halaby T, al Naiemi N, Beishuizen B, Verkooijen R, Ferreira JA, Klont R, et al. Impact of single room design on the spread of multi-drug resistant bacteria in an intensive care unit. Antimicrobial Resistance & Infection Control. 2017 2017/11/15;6(1):117.
5. Decraene V, Phan HTT, George R, Wyllie DH, Akinremi O, Aiken Z, et al. A Large, Refractory Nosocomial Outbreak of Klebsiella pneumoniae Carbapenemase-Producing Escherichia coli Demonstrates Carbapenemase Gene Outbreaks Involving Sink Sites Require Novel Approaches to Infection Control. Antimicrob Agents Chemother. 2018 Dec;62(12).
6. Frakking FNJ, Bril WS, Sinnige JC, Klooster JEV, de Jong BAW, van Hannen EJ, et al. Recommendations for the successful control of a large outbreak of vancomycin-resistant Enterococcus faecium in a non-endemic hospital setting. J Hosp Infect. 2018 Dec;100(4):e216-e25.
7. Otter JA, Burgess P, Davies F, Mookerjee S, Singleton J, Gilchrist M, et al. Counting the cost of an outbreak of carbapenemase-producing Enterobacteriaceae: an economic evaluation from a hospital perspective. Clin Microbiol Infect. 2017 Mar;23(3):188-96.
8. World Health Organisation. Antibiotic Shortages: Magnitude, Causes and Possible Solutions. Oslo2018.
9. Wilkinson E. How precarious antibiotic supplies are putting patients at risk. 2021 3 September 2021]; Available from: https://pharmaceutical-journal.com/article/feature/how-precarious-antibiotic-supplies-are-putting-patients-at-risk
10. HM Government. Tackling antimicrobial resistance 2019–2024: The UK's five-year national action plan. London2019.
11. Gross AE, Johannes RS, Gupta V, Tabak YP, Srinivasan A, Bleasdale SC. The Effect of a Piperacillin/Tazobactam Shortage on Antimicrobial Prescribing and Clostridium difficile Risk in 88 US Medical Centers. Clinical Infectious Diseases. 2017;65(4):613-8.
12. Barber KE, Bell AM, Travis King S, Parham JJ, Stover KR. Impact of piperacillin-tazobactam shortage on meropenem use: implications for antimicrobial stewardship programs. The Brazilian Journal of Infectious Diseases. 2016 2016/11/01/;20(6):631-4.
13. Puzniak L, Dillon R, Palmer T, Collings H, Enstone A. Real-world use of ceftolozane/tazobactam: a systematic literature review. Antimicrobial Resistance & Infection Control. 2021 2021/04/08;10(1):68.
14. Cederwall I ML, Faghihi L, Ali Mohsen L, Yekerusta R. An Investigation of Aspects Affecting Availability and the Health-economical Consequences of Shortages ofVancomycin and Piperacillin/Tazobactam. Uppsala: Uppsala University; 2020.
15. American Society of Hospital Pharmacists (ASHP). Drug Shortages List. 15 August 2021]; Available from: https://www.ashp.org/Drug-Shortages/Current-Shortages/Drug-Shortages-List?page=All
16. Morris DE, Cleary DW, Clarke SC. Secondary Bacterial Infections Associated with Influenza Pandemics. Frontiers in Microbiology. 2017 2017-June-23;8(1041).
17. Madhav N, Oppenheim B, Gallivan M, Mulembakani P, Rubin E, Wolfe N. Pandemics: Risks, Impacts, and Mitigation. In: Jamison DT, Gelband H, Horton S, Jha P, Laxminarayan R, Mock CN, et al., editors. Disease Control Priorities: Improving Health and Reducing Poverty. 3rd ed. Washington DC: The International Bank for Reconstruction and Development / The World Bank; 2017.
18. Department of Health. Use of Antibiotics in an Influenza Pandemic - Scientific Evidence Base Review. London2011.
19. MacIntyre CR, Chughtai AA, Barnes M, Ridda I, Seale H, Toms R, et al. The role of pneumonia and secondary bacterial infection in fatal and serious outcomes of pandemic influenza a(H1N1)pdm09. BMC Infect Dis. 2018 Dec 7;18(1):637.
20. Dhanoa A, Fang NC, Hassan SS, Kaniappan P, Rajasekaram G. Epidemiology and clinical characteristics of hospitalized patients with pandemic influenza A (H1N1) 2009 infections: the effects of bacterial coinfection. Virol J. 2011 Nov 3;8:501.
21. Megiddo I, Drabik D, Bedford T, Morton A, Wesseler J, Laxminarayan R. Investing in antibiotics to alleviate future catastrophic outcomes: What is the value of having an effective antibiotic to mitigate pandemic influenza? Health Econ. 2019 Apr;28(4):556-71.
22. Rawson TM, Moore LSP, Zhu N, Ranganathan N, Skolimowska K, Gilchrist M, et al. Bacterial and Fungal Coinfection in Individuals With Coronavirus: A Rapid Review To Support COVID-19 Antimicrobial Prescribing. Clin Infect Dis. 2020;71(9):2459-68.
23. O'Neill J. Tackling drug-resistant infections globally: Final report and recommendations. London2016.
24. de Kraker MEA, Stewardson AJ, Harbarth S. Will 10 Million People Die a Year due to Antimicrobial Resistance by 2050? PLoS Med. 2016;13(11):e1002184-e.
25. KPMG LLP. The global economic impact of anti-microbial resistance. London2014.
26. Cabinet Office. UK National Risk Register 2020. London2020.
27. World Economic Forum. The Global Risks Report 2018. Geneva2018.

28. Viens AM, Littmann J. Is Antimicrobial Resistance a Slowly Emerging Disaster? Public Health Ethics. 2015;8(3):255-65.
29. Alvarez-Uria G, Gandra S, Mandal S, Laxminarayan R. Global forecast of antimicrobial resistance in invasive isolates of Escherichia coli and Klebsiella pneumoniae. Int J Infect Dis. 2018 Mar;68:50-3.
30. Cravo Oliveira Hashiguchi T, Ait Ouakrim D, Padget M, Cassini A, Cecchini M. Resistance proportions for eight priority antibiotic-bacterium combinations in OECD, EU/EEA and G20 countries 2000 to 2030: a modelling study. Euro Surveill. 2019 May;24(20).
31. Durham LK, Ge M, Cuccia AJ, Quinn JP. Modeling antibiotic resistance to project future rates: quinolone resistance in Escherichia coli. Eur J Clin Microbiol Infect Dis. 2010 Mar;29(3):353-6.
32. Colson AR, Megiddo I, Alvarez-Uria G, Gandra S, Bedford T, Morton A, et al. Quantifying uncertainty about future antimicrobial resistance: Comparing structured expert judgment and statistical forecasting methods. PLOS ONE. 2019;14(7):e0219190.
33. Cubi-Molla P, Mott D, Henderson N, Zamora B, Grobler M, Garau M. Resource Allocation in Public Sector Programmes: Does the Value of a Life Differ Between Governmental Departments? London: Office of Health Economics; 2021.
34. National Institute for Health and Care Excellence. Guide to the methods of technology appraisal 2013. 2013.
35. Hurst M. The Green Book: Central Government Guidance on Appraisal and Evaluation: HM Treasury, London, UK: OGL Press, 2018 (also available online on www. gov. uk/government/publications), ISBN 978-1-912225-57-6. Taylor & Francis; 2019.
36. Skills for Care. The value of adult social care in England. Leeds2021.
37. Bartsch SM, McKinnell JA, Mueller LE, Miller LG, Gohil SK, Huang SS, et al. Potential economic burden of carbapenem-resistant Enterobacteriaceae (CRE) in the United States. Clin Microbiol Infect. 2017 Jan;23(1):48.e9-.e16.
38. Stone SP, Beric V, Quick A, Balestrini AA, Kibbler CC. The effect of an enhanced infection-control policy on the incidence of Clostridium difficile infection and methicillin-resistant Staphyloccocus aureus colonization in acute elderly medical patients. Age Ageing. 1998 Sep;27(5):561-8.
39. Farrington M, Redpath C, Trundle C, Coomber S, Brown NM. Winning the battle but losing the war: methicillin-resistant Staphylococcus aureus (MRSA) infection at a teaching hospital. Qjm. 1998 Aug;91(8):539-48.
40. Selkon JB, Stokes ER, Ingham HR. The role of an isolation unit in the control of hospital infection with methicillin-resistant staphylococci. J Hosp Infect. 1980 Mar;1(1):41-6.
41. Zasowski EJ, Bassetti M, Blasi F, Goossens H, Rello J, Sotgiu G, et al. A Systematic Review of the Effect of Delayed Appropriate Antibiotic Treatment on the Outcomes of Patients With Severe Bacterial Infections. CHEST. 2020;158(3):929-38.
42. Yang M, Gao H, Chen J, Xu X, Tang L, Yang Y, et al. Bacterial coinfection is associated with severity of avian influenza A (H7N9), and procalcitonin is a useful marker for early diagnosis. Diagnostic Microbiology and Infectious Disease. 2016 2016/02/01/;84(2):165-9.
43. Scientific Pandemic Influenza Group on Modelling. SPI-M Modelling Summary. London2018.
44. Fan VY, Jamison DT, Summers LH. Pandemic risk: how large are the expected losses? Bull World Health Organ. 2018;96(2):129-34.
45. Chisti MJ, Harris JB, Carroll RW, Shahunja KM, Shahid ASMSB, Moschovis PP, et al. Antibiotic-Resistant Bacteremia in Young Children Hospitalized With Pneumonia in Bangladesh Is Associated With a High Mortality Rate. Open Forum Infectious Diseases. 2021;8(7).
46. Garrison LP, Jr., Zamora B, Li M, Towse A. Augmenting Cost-Effectiveness Analysis for Uncertainty: The Implications for Value Assessment-Rationale and Empirical Support. J Manag Care Spec Pharm. 2020 Apr;26(4):400-6.
47. Rowen D, Dixon S, Hernandez M, Mukuria C. ESTIMATING INFORMAL CARE EFFECTS ASSOCIATED WITH EQ-5D TARIFF VALUES FOR USE IN ECONOMIC EVALUATION: Policy Research Unit in Economic Evaluation of Health & Care Interventions (EEPRU); 2013.
48. Eurostat. Total wages and salaries. 2021.
49. Saloniki E, Nizalova O, Malisauskaite G, Forder J. The impact of formal care on informal care for people over 75 in England2019.
50. Soares M, Claxton K. Appendix A: Displacement by ICD code. 6 October 2021]; Available from: https://www.york.ac.uk/media/che/documents/Appendix%20A%20(Displacement%20by%20ICD%20code).pdf
51. Claxton K, Martin S, Soares M, Rice N, Spackman E, Hinde S, et al. Methods for the estimation of the National Institute for Health and Care Excellence cost-effectiveness threshold. Health Technol Assess. 2015 Feb;19(14):1-503, v-vi.
52. Office for National Statistics. Average weekly earnings in Great Britain: August 2021. 2021 [cited 26 August 2021; Available from: https://www.ons.gov.uk/employmentandlabourmarket/peopleinwork/employmentandemployeetypes/bulletins/averageweeklyearningsingreatbritain/latest
53. Sadique Z, Lopman B, Cooper BS, Edmunds WJ. Cost-effectiveness of Ward Closure to Control Outbreaks of Norovirus Infection in United Kingdom National Health Service Hospitals. J Infect Dis. 2016 Feb 1;213 Suppl 1:S19-26.
54. Lopman BA, Reacher MH, Vipond IB, Hill D, Perry C, Halladay T, et al. Epidemiology and cost of nosocomial gastroenteritis, Avon, England, 2002–2003. Emerging infectious diseases. 2004;10(10):1827.
55. Barber KE, Bell AM, Travis King S, Parham JJ, Stover KR. Impact of piperacillin-tazobactam shortage on meropenem use: implications for antimicrobial stewardship programs. Braz J Infect Dis. 2016 Nov - Dec;20(6):631-4.
56. Bosso JA, Kokko H. Assessment of the impact of an antibiotic shortage: patient outcomes and costs. Hospital Pharmacy. 2004;39(1):43-7.
57. Burns A, Van der Mensbrugghe D, Timmer H. Evaluating the economic consequences of avian influenza. 2006.

58. Marchetti A, Rossiter R. Economic burden of healthcare-associated infection in US acute care hospitals: societal perspective. J Med Econ. 2013 Dec;16(12):1399-404.
59. Shreve J, Van Den Bos J, Gray T, Halford M, Rustagi K, Ziemkiewicz E. The economic measurement of medical errors. Society of Actuaries. 2010. 2010.
60. Vrijens F, Hulstaert F, Devriese S, van de Sande S. Hospital-acquired infections in Belgian acute-care hospitals: an estimation of their global impact on mortality, length of stay and healthcare costs. Epidemiol Infect. 2012 Jan;140(1):126-36.
61. World Bank Group. Drug Resistant Infections: A Threat to our Economic Future. Washington DC2017.
62. Public Health England. AMR local indicators - Fingertips profile. 2018 22 Feb 2019]; Available from: https://fingertips.phe.org.uk/profile/amr-local-indicators
63. Brassel S, Neri M, Schirrmacher H, Steuten L. The Value of Vaccines in Maintaining Health System Capacity in England. London2021.
64. Moss JWE, Davidson C, Mattock R, Gibbons I, Mealing S, Carroll S. Quantifying the direct secondary health care cost of seasonal influenza in England. BMC Public Health. 2020 2020/09/29;20(1):1464.
65. UK Government. Coronavirus (COVID-19) in the UK. 2021 3 June 2021];
66. Welsh Government. Together for Health – A Delivery Plan for the Critically Ill. Cardiff2013.
67. NHS Digital. Hospital Admitted Patient Care Activity 2018-19. 6 October 2021]; Available from: https://digital.nhs.uk/data-and-information/publications/statistical/hospital-admitted-patient-care-activity/2018-19
68. Soriano A, Carmeli Y, Omrani AS, Moore LSP, Tawadrous M, Irani P. Ceftazidime-Avibactam for the Treatment of Serious Gram-Negative Infections with Limited Treatment Options: A Systematic Literature Review. Infect Dis Ther. 2021 Aug 11:1-46.
69. Tumbarello M, Viale P, Viscoli C, Trecarichi EM, Tumietto F, Marchese A, et al. Predictors of mortality in bloodstream infections caused by Klebsiella pneumoniae carbapenemase-producing K. pneumoniae: importance of combination therapy. Clin Infect Dis. 2012 Oct;55(7):943-50.
70. Gordon J, Darlington O, McEwan P, Lumley M, Taie A, Hicks M, et al. Estimating the Value of New Antimicrobials in the Context of Antimicrobial Resistance: Development and Application of a Dynamic Disease Transmission Model. PharmacoEconomics. 2020 2020/08/01;38(8):857-69.
